# PyIOmica: longitudinal omics analysis and trend identification

**DOI:** 10.1093/bioinformatics/btz896

**Published:** 2019-11-28

**Authors:** Sergii Domanskyi, Carlo Piermarocchi, George I Mias

**Affiliations:** 1 Department of Physics and Astronomy, East Lansing, MI 48824, USA; 2 Department of Biochemistry and Molecular Biology, East Lansing, MI 48824, USA; 3 Institute for Quantitative Health Science and Engineering, Michigan State University, East Lansing, MI 48824, USA

## Abstract

**Summary:**

PyIOmica is an open-source Python package focusing on integrating longitudinal multiple omics datasets, characterizing and categorizing temporal trends. The package includes multiple bioinformatics tools including data normalization, annotation, categorization, visualization and enrichment analysis for gene ontology terms and pathways. Additionally, the package includes an implementation of visibility graphs to visualize time series as networks.

**Availability and implementation:**

PyIOmica is implemented as a Python package (pyiomica), available for download and installation through the Python Package Index (https://pypi.python.org/pypi/pyiomica), and can be deployed using the Python import function following installation. PyIOmica has been tested on Mac OS X, Unix/Linux and Microsoft Windows. The application is distributed under an MIT license. Source code for each release is also available for download on Zenodo (https://doi.org/10.5281/zenodo.3548040).

**Supplementary information:**

Supplementary data are available at *Bioinformatics*

## 1 Introduction

As sequencing costs continue to drop, systems biology based on large omics datasets is rapidly expanding its scope. In particular, time series obtained from multi-omics datasets are becoming more and more affordable (Chen *et al.*, 2012; Garrett-Bakelman *et al.*, 2019; Price *et al.*, 2017). The analysis of time series can have broad implications for precision medicine applications, since longitudinal data capture the dynamically changing collective microscopic behavior of molecular components in the body, reflecting the physiological state of a patient. There are many bioinformatics tools aiming at multimodal omics data integration (Pinu *et al.*, 2019). Specifically, Bioconductor (Gentleman *et al.*, 2004), Galaxy(Afgan *et al.*, 2018), GenePattern (Reich *et al.*, 2006), Biopython (Cock *et al.*, 2009), Pathomx (Fitzpatrick *et al.*, 2014), SECIMTools (Kirpich *et al.*, 2018) and more. Although multiple coding paradigms are used in bioinformatics, R and Python are essentially the lingua francas for data science analysis, where the open-source appeal and growing online community support are particularly helpful in developing a dedicated user base.

Here we introduce PyIOmica, an open source Python package, for analyzing longitudinal omics datasets, such as transcriptomics, proteomics, metabolomics etc., which includes multiple tools for processing multi-modal mapped data, characterizing time series in terms of periodograms and autocorrelations, categorizing temporal behavior, visualizing visibility graphs and testing data for gene ontology and pathway enrichment. PyIOmica includes optimized new algorithms adapted from MathIOmica (Mias *et al.*, 2016; which runs on the proprietary Mathematica platform), now made available as Python open source code for all users, and additionally expands extensively graphical utilities for visualization of categorized temporal data, and network representation of time series. To our knowledge, there are no tools with the functionality of PyIOmica currently available in Python.

## 2 Materials and methods

### 2.1 Overview and codebase

PyIOmica provides a complete workflow for time series processing, illustrated in the Supplementary Figure S1. The modular nature of PyIOmica allows for smooth integration with any future and existing Python tools. With PyIOmica, any results can be visualized, exported and analyzed for gene enrichment by means of a user-friendly Python interface. PyIOmica’s codebase is a single Python module containing multiple groups of functions designed for annotations and enumerations, pre- and post-processing, clustering-related purposes, visualizations (heatmaps and categorization), normal and horizontal visibility graphs generation and other core and utility components. Installation is simply performed using pip install pyiomica, and package dependencies are automatically addressed directly from Python package index (PyPI). Function documentation is embedded in the module, and is easily accessible at runtime (and also at https://pyiomica.readthedocs.io). Data structures and implementation are described in Supplementary Material.

An extensive set of PyIOmica pre-processing functions enables filtering low-quality signals, tagging missing or low values, normalization, standardization, merging and comparison of the datasets. The post-processing functions, such as temporal trends categorization of power spectrum and spikes, are built on using the SciPy and scikit-learn Python toolkits. Additional functionality includes gene ontology (GO) and Kyoto Encyclopedia of Genes and Genomes (KEGG) pathway enrichment analyses for both non-temporal data, as well as for clusters identified through the automated time series categorization.

Temporal trends are automatically discovered using periodogram and autocorrelation calculations based on a Lomb-Scargle transformation algorithm (Mias *et al.*, 2016), which properly accounts for missing points and/or unevenly sampled data. The periodogram is used to identify each time series’ underlying dominant frequencies. Autocorrelations are also used to identify how measured intensities within each time series may depend on previous measurements, by correlating a time series with delayed versions of itself. Signals showing statistically significant trends are identified for downstream analysis. Multiple omics (genes, proteins and metabolites) that show similar trends in time are identified by clustering, and can be biologically evaluated through pathway and GO analyses.

### 2.2 Visibility graphs and visualization

Recent work on characterizing complex events focuses on using network/graph methodology that can capture non-linear behavior (Lacasa *et al.*, 2008). Time series are transformed into networks that conserve their topology, and allow the identification of varying temporal structures. We represent each timepoint in a series as a node. Then, for any timepoint pair with intensities $X(t_{\mu }),\;X(t_{\nu })$ at times $t_{\mu }$ and $t_{\nu }$ respectively, we can have an edge if for any other timepoint $t_{\alpha }$, such that $\left(t_{\mu }<t_{\alpha }<t_{\nu }\right)$ we have $X(t_{\alpha })<X(t_{\nu })+(X(t_{\mu })-X(t_{\nu }))\frac{t_{\nu }-t_{\alpha }}{t_{\nu }-t_{\mu }}$. Representing the intensities as bars, this is equivalent to connecting the top of each bar to another top if there is a direct line-of-sight to that top. The resulting *visibility graph* has characteristics that reflect the equivalent time series temporal structure and can be used to identify trends. The shortest path identifies nodes (i.e. timepoints) that display high intensity, and thus dominate the global signal profile, are robust to noise, and are likely drivers of the global temporal behavior. A biological event deviating from baseline is likely to appear in one or more nodes within the shortest path.

**Fig. 1.**
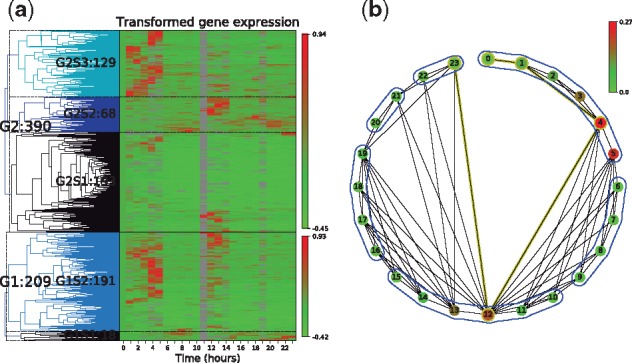
Example PyIOmica data visualization. (**a**) Dendrogram with heatmap of automatically categorized longitudinal gene expression data. Autocorrelations are used to identify temporal trends in the data. Subgroups are determined based on similar collective behavior over time. (**b**) Visibility graph of median signal intensity from group G1S2 from (a)

PyIOmica uses Matplotlib plotting functions to visualize histograms, dendrograms, heatmaps and visibility graphs. Figure 1a shows example RNA-sequencing gene expression data from a 24-h time series, clustered into two groups based on autocorrelations. Subgroups were determined from the gene expression in each autocorrelation group. The data from Group 1, Subgroup 2 containing 191 genes is visualized in Figure 1b as a visibility graph on a circular layout. Temporal events are detected and indicated with solid blue lines encompassing groups of points, or communities. Additional examples are provided in the PyIOmica documentation (Supplementary Material, using data that are provided with the PyIOmica Zenodo software release (under docs/examples)).

## 3 Conclusion

The open source PyIOmica Python package characterizes time series from multiple omics and categorizes temporal trends with a streamlined automated pipeline based on spectral analysis. PyIOmica also offers broad bioinformatics functionality, including clustering, visualization and enrichment, and extends previous developments (Mias *et al.*, 2016) to an open-source, community-accessible platform for data science. We anticipate future versions of PyIOmica to utilize its codebase flexibility to expand its bioinformatics tools for genomic as well as differential omics analyses, and graph construction and characterization.

## Funding

This work was supported by the Translational Research Institute for Space Health through National Aeronautics and Space Administration (NASA) Cooperative Agreement NNX16AO69A.

*Conflict of Interest:* G.M. has consulted for Colgate-Palmolive North America. C.P. owns equity in Salgomed, Inc. S.D. reports no potential confict of interest.

## Supplementary Material

btz896_Supplementary_Data
