# Supplementary material for: PyIOmica: longitudinal omics analysis and trend identification
Source: Bioinformatics. 2019 Nov 28;36(7):2306–7. doi: 10.1093/bioinformatics/btz896 (PMC7141865; doi:10.1093/bioinformatics/btz896)
Supplement: btz896_Supplementary_Data [file btz896_supplementary_data.zip › btz896-suppl_data/Pyiomica Supplementary Material.pdf]

Supplementary Material: PyIOmica: Longitudinal Omics Analysis and Trend Identification

Sergii Domanskyi [1], Carlo Piermarocchi, [1] and George I. Mias [1,2,3,*]

[1]Physics and Astronomy, Michigan State University, East Lansing, MI 48824, USA,
[2] Biochemistry and Molecular Biology, Michigan State University, East Lansing, MI 48824, USA and
[3]Institute for Quantitative Health Science and Engineering, Michigan State University MI 48824, USA.

* To whom correspondence should be addressed: gmias@msu.edu.

1 SUPPLEMENTARY TEXT

1.1 PyIOmica information

PyIOmica is a Python package released under an MIT license through the Python Package Index. The current version used in this manuscript is 1.1.1 (10/26/19). The documentation with examples is available at Read the Docs: https://pyiomica.readthedocs.io/.

1.2 PyIOmica Installation Instructions

The following installation instructions have been tested on Mac OS 10.14.5+, Windows 10, and Ubuntu using the Anaconda Python Distribution.

1.2.1 Pre-Installation Requirements

To install PyIOmica on any platform you need Python version 3.7 or higher

1.2.2 Installation

To install the current release from PyPI (Python Package Index) use pip:

```
$ pip install pyiomica
```

Alternatively, you can install directly from github using:

```
$ pip install git+https://github.com/gmiaslab/pyiomica/
```

1.3 Running PyIOmica

After installation you can run PyIOmica in the Python REPL:

```
>>> import pyiomica
```

Import any submodule from PyIOmica, e.g.:

```
>>> from pyiomica import categorizationFunctions
```

2 SUPPLEMENTARY FIGURES

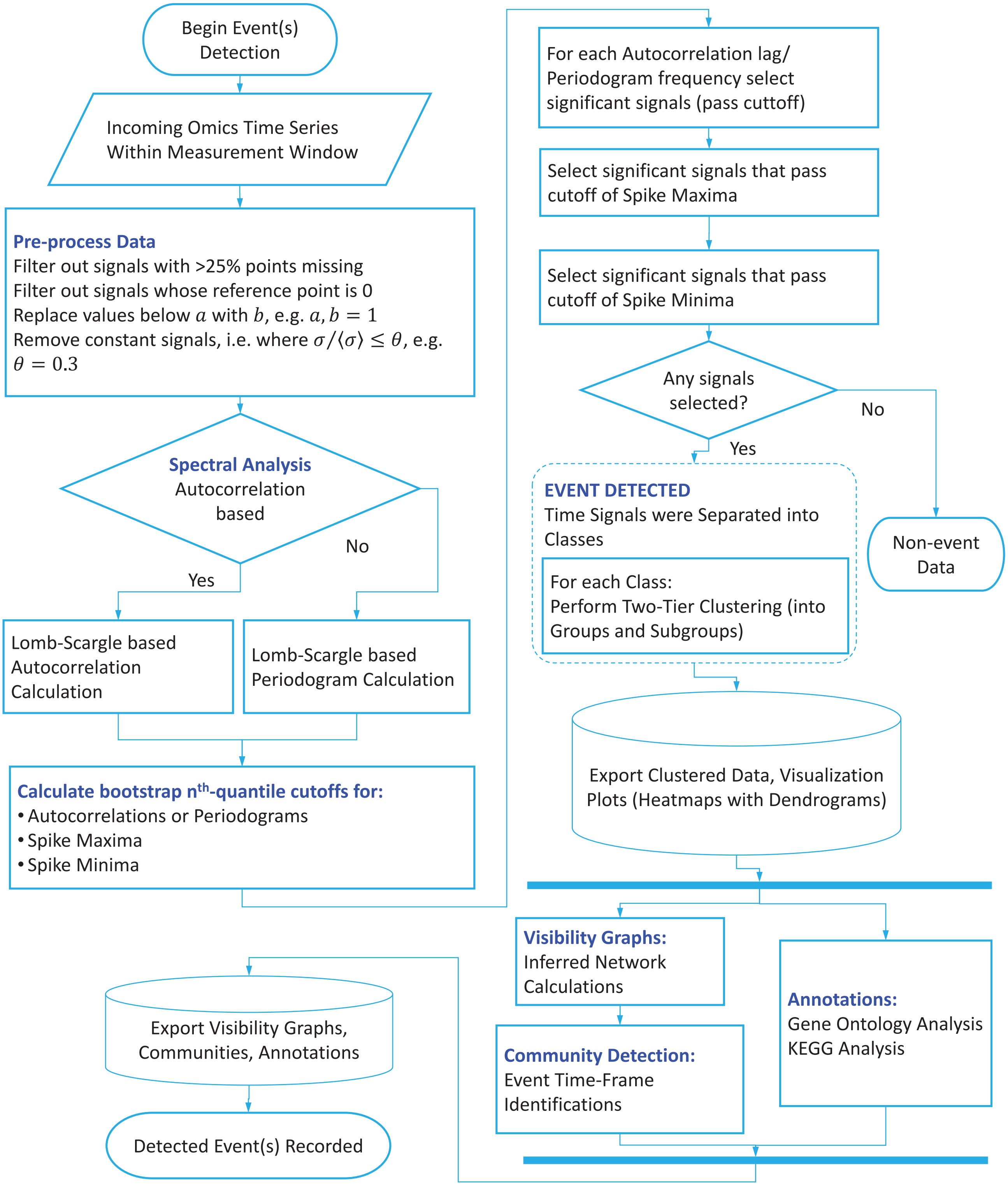

Figure S1. PyIOmica Categorization Scheme. PyIOmica analyzes multi-omics time series analysis utilizing spectral analysis, bootstrap methods for statistics, community detection in clustering and visibility graphs, and annotates results with Gene Ontology and pathway analyses.

3 DATA STRUCTURE AND ANALYSES

We utilize the fast, robust, and versatile functionality of Python Pandas' DataFrame, a two-dimensional size-mutable data structure that allows for multi-level hierarchical indexing (MultiIndex) along both axes (rows and columns). We extend Pandas' DataFrame by encapsulating various data processing functions in our Extended DataFrame.

Input data from files of various formats, such as CSV, TSV, XLSX etc. are imported by user of PyIOmica and converted to our Extended DataFrame for processing, aggregation and storage. MathIOmica data types can be also imported by means of function `readMathIOmicaData` in submodule `utilityFunctions`.

PyIOmica uses multi-level hierarchical indexing to store metadata. For example, gene-specific information in a transcriptome dataset can be encoded by adding new row `MultiIndex` levels, and sample-specific information can be added using column `MultiIndex` levels. For storage and exchange, data are written using the Hierarchical Data Format (HDF5), which is essential when large amounts of data are supplied. The HDF5 files are easily accessed using `pandas.HDFStore`, which utilizes `PyTables`, or using `h5py` to read/write `NumPy` arrays.

PyIOmica introduces a Python implementation of the standard normal and horizontal visibility graphs, whose adjacency matrices are calculated using two methods: a `Numba` JIT-accelerated approach scalable to multiple CPUs, and a `NumPy`-accelerated approach, which is preferred for large datasets but not readily scalable beyond a few CPUs. The adjacency matrix is then converted to a `NetworkX` graphs for visualization and analysis.

4 ADDENDUM: PYIOMICA FUNCTION REFERENCES

4.1 Documentation

Documentation for PyIOmica is built-in and is available through the help command, for the complete documentation:

```
>>> help(pyiomica)
```

Or per function. For example:

```
>>> help(function)
```

The per-function documentation is also available in the PyIOmica Manual, which is included below. Additional examples are available as a Jupyter notebook. The documentation is provided in the `docs` directory distributed with the PyIOmica release - see GitHub and Zenodo for latest versions.

pyiomica Documentation

Release 1.1.1

S. Domanskyi, C. Piermarocchi, G. Mias

Oct 26, 2019

Contents:

1 Overview **3**

1.1 Versions 3
1.2 Documentation 4
1.3 Additional information 4
1.4 Licensing 4
1.5 Contact information 4
1.6 Funding 4

2 Installation **5**

2.1 Pre-Installation Requirements 5
2.2 Installation Instructions 5
2.3 Running PyIOmica 5

3 Functionality **7**

3.1 Global variables 7
3.2 Categorization functions 8
3.3 Enrichment analyses functions 11
3.4 Extended DataFrame and data-processing functions 19
3.5 Clustering functions 26
3.6 Visibility graph preparation functions 29
3.7 Visualization functions 30
3.8 Utility functions 35

4 Dependencies **37**

5 Included data **39**

6 Examples **41**

6.1 Enrichment report 41
6.2 Import of MathIOmica Objects 42
6.3 Clustering object export example 43
6.4 GO Analysis examples 43
6.5 KEGG Analysis examples 45
6.6 Visibility Graph examples 47
6.7 Extended DataFrame 53
6.8 Time Series Categorization 55

7 Indices and tables **61**

Python Module Index **63**

Index **65**

This documentation describes PyIOmica, a Python package that provides bioinformatics utilities for analyzing (dynamic) omics datasets. PyIOmica extends MathIOmica usage to Python and implements new visualizations and computational tools for graph analyses.

CHAPTER 1

Overview

An open source Python package, for analyzing longitudinal omics datasets, which includes multiple tools for processing of multi-modal mapped data, characterizing time series in terms of periodograms and autocorrelations, classifying temporal behavior, visualizing visibility graphs, and testing data for gene ontology and pathway enrichment. PyIOmica includes optimized new algorithms adapted from MathIOmica (which runs on the proprietary Mathematica platform), now made available as Python open source code for all users, and additionally expands extensively graphical utilities for visualization of classified temporal data, and network representation of time series.

1.1 Versions

- 1.1.1
  - Updated examples Jupyter notebook.
  - Small typographical fixes.
- 1.1.0
  - Restructured all modules.
  - Developed ReadTheDocs documentation.
- 1.0.2
  - Updated setup dependencies for pip compatibility.
- 1.0.1
  - Updated setup dependencies.
- 1.0.0
  - First Release.

1.2 Documentation

Documentation for PyIOmica is built-in and is available through the help() functionality in Python or online at https://pyiomica.readthedocs.io.

1.3 Additional information

- PyIOmica is a multi-omics analysis framework distributed as a Python package that aims to assist in bioinformatics.
- The most current version of the package is maintained at https://github.com/gmiaslab/pyiomica
- News are distributed via twitter (@mathiomica)

1.4 Licensing

PyIOmica is released under an MIT License. Please also consult the folder LICENSES distributed with PyIOmica regarding Licensing information for use of external associated content.

1.5 Contact information

- Contributors: Sergii Domanskyi, Carlo Piermarocchi, George I. Mias.
- G.MiasLab (https://georgemias.org)
- e-mail: gmiaslab@gmail.com
- twitter: @gmiaslab

1.6 Funding

PyIOmica development and associated research were supported by the Translational Research Institute for Space Health through NASA Cooperative Agreement NNX16AO69A (Project Number T0412, PI: Mias). The content is solely the responsibility of the authors and does not necessarily represent the official views of the supporting funding agencies.

CHAPTER 2

Installation

2.1 Pre-Installation Requirements

To install PyIOmica on any platform you need Python version 3.7 or higher

2.2 Installation Instructions

1. To install the current release from PyPI (Python Package Index) use pip:

```
pip install pyiomica
```

2. Alternatively, you can install directly from github using:

```
pip install git+https://github.com/gmiaslab/pyiomica/
```

2.3 Running PyIOmica

After installation run:

```
>>> import pyiomica
```

CHAPTER 3

Functionality

3.1 Global variables

Submodule **pyiomica.globalVariables**

This module contains global constants used in PyIOmica. Some of the modules, classes and functions are imported in this module.

Data

ConstantGeneDictionary	ConstantGeneDictionary is a global gene/protein dictionary variable typically created by GetGeneDictionary.
ConstantPyIOmicaDataDirectory	ConstantPyIOmicaDataDirectory is a global variable pointing to the PyIOmica data directory.
ConstantPyIOmicaExampleVideosDirectory	ConstantPyIOmicaExampleVideosDirectory is a global variable pointing to the PyIOmica example videos directory.
ConstantPyIOmicaExamplesDirectory	ConstantPyIOmicaExamplesDirectory is a global variable pointing to the PyIOmica example data directory.
PackageDirectory	Package directory
printPackageGlobalDefaults	Whether to print package global defaults listed in this module

printPackageGlobalDefaults = False
Whether to print package global defaults listed in this module

PackageDirectory = '/home/docs/checkouts/readthedocs.org/user_builds/pyiomica/checkouts/lat
Package directory

ConstantPyIOmicaDataDirectory = '/home/docs/checkouts/readthedocs.org/user_builds/pyiomica/
ConstantPyIOmicaDataDirectory is a global variable pointing to the PyIOmica data directory.

ConstantPyIOmicaExamplesDirectory = '/home/docs/checkouts/readthedocs.org/user_builds/pyio
ConstantPyIOmicaExamplesDirectory is a global variable pointing to the PyIOmica example data directory.

ConstantPyIOmicaExampleVideosDirectory = '/home/docs/checkouts/readthedocs.org/user_builds/
ConstantPyIOmicaExampleVideosDirectory is a global variable pointing to the PyIOmica example videos directory.

ConstantGeneDictionary = None
ConstantGeneDictionary is a global gene/protein dictionary variable typically created by GetGeneDictionary.

3.2 Categorization functions

Submodule **pyiomica.categorizationFunctions**

Example of use of a function described in this module:

```
# import the package and its mudule Categorization functions
import pyiomica as pio
from pyiomica import categorizationFunctions as cf

# Location of this example data
dir = pio.ConstantPyIOmicaExamplesDirectory

# Unzip sample data
with pio.zipfile.ZipFile(pio.os.path.join(dir, 'SLV.zip'), "r") as zipFile:
    zipFile.extractall(path=dir)

# Process sample dataset SLV Hourly 1
dataName = 'SLV_Hourly1TimeSeries'
saveDir = pio.os.path.join('results', dataName, '')
dataDir = pio.os.path.join(dir, 'SLV')
df_data = pio.pd.read_csv(pio.os.path.join(dataDir, dataName + '.csv'), index_col=[0,
↪1,2], header=0)
cf.calculateTimeSeriesCategorization(df_data, dataName, saveDir,␣
↪NumberOfRandomSamples = 10**4)
cf.clusterTimeSeriesCategorization(dataName, saveDir)
cf.visualizeTimeSeriesCategorization(dataName, saveDir)
```

One of the figures generated by `visualizeTimeSeriesCategorization` is shown below:

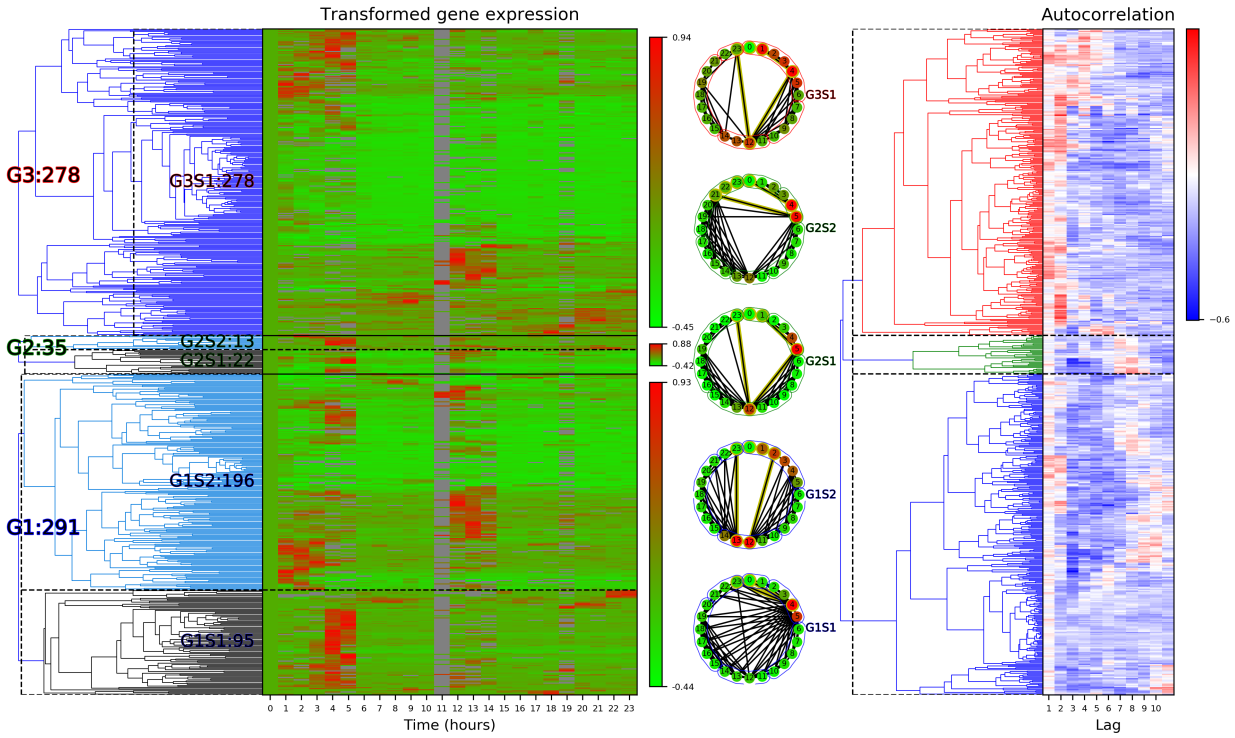

Categorization functions

Functions

`calculateTimeSeriesCategorization`(df_data, ...)	Time series classification.
`clusterTimeSeriesCategorization`(dataName, ...)	Visualize time series classification.
`visualizeTimeSeriesCategorization`(dataName, ...)	Visualize time series classification.

calculateTimeSeriesCategorization(*df_data*, *dataName*, *saveDir*, *hdf5fileName=None*, *p_cutoff=0.05*, *fraction=0.75*, *constantSignalsCutoff=0.0*, *lowValuesToTag=1.0*, *lowValuesToTagWith=1.0*, *NumberOfRandomSamples=100000*, *NumberOfCPUs=4*, *referencePoint=0*, *autocorrelationBased=True*, *calculateAutocorrelations=False*, *calculatePeriodograms=False*, *preProcessData=True*)

Time series classification.

Parameters:

df_data: pandas.DataFrame Data to process

dataName: str Data name, e.g. "myData_1"

saveDir: str Path of directories poining to data storage

hdf5fileName: str, Default None Preferred hdf5 file name and location

p_cutoff: float, Default 0.05 Significance cutoff signals selection

fraction: float, Default 0.75 Fraction of non-zero point in a signal

constantSignalsCutoff: float, Default 0. Parameter to consider a signal constant

lowValuesToTag: float, Default 1. Values below this are considered low

lowValuesToTagWith: float, Default 1. Low values to tag with

NumberOfRandomSamples: int, Default 105** Size of the bootstrap distribution to generate

NumberOfCPUs: int, Default 4 Number of processes allowed to use in calculations

referencePoint: int, Default 0 Reference point

autocorrelationBased: boolean, Default True Whether Autocorrelation of Frequency based

calculateAutocorrelations: boolean, Default False Whether to recalculate Autocorrelations

calculatePeriodograms: boolean, Default False Whether to recalculate Periodograms

preProcessData: boolean, Default True Whether to preprocess data, i.e. filter, normalize etc.

Returns: None

Usage: calculateTimeSeriesCategorization(df_data, dataName, saveDir)

clusterTimeSeriesCategorization(*dataName*, *saveDir*, *numberOfLagsToDraw=3*, *hdf5fileName=None*, *exportClusteringObjects=False*, *writeClusteringObjectToBinaries=True*, *autocorrelationBased=True*)

Visualize time series classification.

Parameters:

dataName: str Data name, e.g. "myData_1"

saveDir: str Path of directories poining to data storage

numberOfLagsToDraw: int, Default 3 First top-N lags (or frequencies) to draw

hdf5fileName: str, Default None HDF5 storage path and name

exportClusteringObjects: boolean, Default False Whether to export clustering objects to xlsx files

writeClusteringObjectToBinaries: boolean, Default True Whether to export clustering objects to binary (pickle) files

autocorrelationBased: boolean, Default True Whether to label to print on the plots

Returns: None

Usage: clusterTimeSeriesClassification('myData_1', '/dir1/dir2/')

visualizeTimeSeriesCategorization(*dataName*, *saveDir*, *numberOfLagsToDraw=3*, *autocorrelationBased=True*)

Visualize time series classification.

Parameters:

dataName: str Data name, e.g. "myData_1"

saveDir: str Path of directories poining to data storage

numberOfLagsToDraw: boolean, Default 3 First top-N lags (or frequencies) to draw

autocorrelationBased: boolean, Default True Whether autocorrelation or frequency based

Returns: None

Usage: visualizeTimeSeriesClassification('myData_1', '/dir1/dir2/')

3.3 Enrichment analyses functions

Submodule **pyiomica.enrichmentAnalyses**

Example of use of a function described in this module:

```
import pyiomica as pio

# Import functions necessary for this demo
from pyiomica.enrichmentAnalyses import GOAnalysis, ExportEnrichmentReport

# Specify a directory for output
EnrichmentOutputDirectory = pio.os.path.join('results','EnrichmentOutputDirectory')

# Let's do a GO analysis for a group of genes, annotated with their "Gene Symbol":
goExample1 = GOAnalysis(["TAB1", "TNFSF13B", "MALT1", "TIRAP", "CHUK",
                         "TNFRSF13C", "PARP1", "CSNK2A1", "CSNK2A2", "CSNK2B", "LTBR",
                         "LYN", "MYD88", "GADD45B", "ATM", "NFKB1", "NFKB2", "NFKBIA",
                         "IRAK4", "PIAS4", "PLAU"])

# Export enrichment results in to .xlsx file
ExportEnrichmentReport(goExample1,
                       AppendString='goExample1',
                       OutputDirectory=EnrichmentOutputDirectory + 'GOAnalysis/')
```

Note: Function `ExportEnrichmentReport` generates ".xlsx" file, described in *Examples*.

Annotations and Enumerations

Functions

BenjaminiHochbergFDR(pValues[, . . .])	HypothesisTesting BenjaminiHochbergFDR correction
ExportEnrichmentReport(data[, AppendString, . . .])	Export results from enrichment analysis to Excel spreadsheets.
GOAnalysis(data[, GetGeneDictionaryOptions, . . .])	Calculate input data over-representation analysis for Gene Ontology (GO) categories.
GOAnalysisAssigner([PyIOmicaDataDirectory, . . .])	Download and create gene associations and restrict to required background set.
GeneTranslation(InputList, TargetIDList, . . .)	Use geneDictionary to convert inputList IDs to different annotations as indicated by targetIDList.
GetGeneDictionary([geneUCSCTable, . . .])	Create an ID/accession dictionary from a UCSC search - typically of gene annotations.
KEGGAnalysis(data[, AnalysisType, . . .])	Calculate input data over-representation analysis for KEGG: Kyoto Encyclopedia of Genes and Genomes pathways.
KEGGAnalysisAssigner([. . .])	Create KEGG: Kyoto Encyclopedia of Genes and Genomes pathway associations, restricted to required background set, downloading the data if necessary.
KEGGDictionary([PyIOmicaDataDirectory, . . .])	Create a dictionary from KEGG: Kyoto Encyclopedia of Genes and Genomes terms - typically association of pathways and members therein.
MassDictionary([PyIOmicaDataDirectory])	Load PyIOmica's current mass dictionary.

Continued on next page

Table 3 – continued from previous page

MassMatcher(data, accuracy[, ...])	Assign putative mass identification to input data based on monoisotopic mass (using PyIOmica's mass dictionary).
OBOGODictionary([FileURL, ImportDirectly, ...])	Generate Open Biomedical Ontologies (OBO) Gene Ontology (GO) vocabulary dictionary.
internalAnalysisFunction(data, multiCorr, ...)	Analysis for Multi-Omics or Single-Omics input list The function is used internally and not intended to be used directly by user.
obtainConstantGeneDictionary(GeneDictionary, ...)	Obtain gene dictionary - if it exists can either augment with new information or Species or create new, if not exist then create variable.

internalAnalysisFunction (*data*, *multiCorr*, *MultipleList*, *OutputID*, *InputID*, *Species*, *totalMembers*, *pValueCutoff*, *ReportFilterFunction*, *ReportFilter*, *TestFunction*, *HypothesisFunction*, *FilterSignificant*, *AssignmentForwardDictionary*, *AssignmentReverseDictionary*, *prefix*, *infoDict*)

Analysis for Multi-Omics or Single-Omics input list The function is used internally and not intended to be used directly by user.

Usage: Intended for internal use

OBOGODictionary (*FileURL='http://purl.obolibrary.org/obo/go/go-basic.obo'*, *ImportDirectly=False*, *PyIOmicaDataDirectory=None*, *OBOFile='goBasicObo.txt'*)

Generate Open Biomedical Ontologies (OBO) Gene Ontology (GO) vocabulary dictionary.

Parameters:

FileURL: str, Default "http://purl.obolibrary.org/obo/go/go-basic.obo" Provides the location of the Open Biomedical Ontologies (OBO) Gene Ontology (GO) file in case this will be downloaded from the web

ImportDirectly: boolean, Default False Import from URL regardles is the file already exists

PyIOmicaDataDirectory: str, Default None Path of directories to data storage

OBOFile: str, Default "goBasicObo.txt" Name of file to store data in (file will be zipped)

Returns:

dictionary Dictionary of definitions

Usage: OBODict = OBOGODictionary()

GetGeneDictionary (*geneUCSCTable=None*, *UCSCSQLString=None*, *UCSCSQLSelectLabels=None*, *ImportDirectly=False*, *Species='human'*, *KEGGUCSCSplit=[True, 'KEGG Gene ID']*)

Create an ID/accession dictionary from a UCSC search - typically of gene annotations.

Parameters:

geneUCSCTable: str, Default None Path to a geneUCSCTable file

UCSCSQLString: str, Default None An association to be used to obtain data from the UCSC Browser tables. The key of the association must match the Species option value used (default: human). The value for the species corresponds to the actual MySQL command used

UCSCSQLSelectLabels: str, Default None An association to be used to assign key labels for the data improted from the UCSC Browser tables. The key of the association must match the Species option value used (default: human). The value is a multi component string list corresponding to the matrices in the data file, or the tables used in the MySQL query provided by UCSCSQLString

ImportDirectly: boolean, Default False Import from URL regardles is the file already exists

Species: str, Default "human" Species considered in the calculation, by default corresponding to human

KEGGUCSCSplit: list, Default [True,"KEGG Gene ID"] Two component list, {True/False, label}. If the first component is set to True the initially imported KEGG IDs, identified by the second component label, are split on + string to fix nomenclature issues, retaining the string following +

Returns:

dictionary Gene dictionary

Usage: geneDict = GetGeneDictionary()

GOAnalysisAssigner(*PyIOmicaDataDirectory=None, ImportDirectly=False, BackgroundSet=[], Species='human', LengthFilter=None, LengthFilterFunction=<ufunc 'greater_equal'>, GOFileName=None, GOFileColumns=[2, 5], GOURL='http://current.geneontology.org/annotations/'*)

Download and create gene associations and restrict to required background set.

Parameters:

PyIOmicaDataDirectory: str, Default None The directory where the default package data is stored

ImportDirectly: boolean, Default False Import from URL regardles is the file already exists

BackgroundSet: list, Default [] Background list to create annotation projection to limited background space, involves considering pathways/groups/sets and that provides a list of IDs (e.g. gene accessions) that should be considered as the background for the calculation

Species: str, Default "human" Species considered in the calculation, by default corresponding to human

LengthFilterFunction: function, Default np.greater_equal Performs computations of membership in pathways/ontologies/groups/sets, that specifies which function to use to filter the number of members a reported category has compared to the number typically provided by LengthFilter

LengthFilter: int, Default None Argument for LengthFilterFunction

GOFileName: str, Default None The name for the specific GO file to download from the GOURL if option ImportDirectly is set to True

GOFileColumns: list, Default [2, 5] Columns to use for IDs and GO:accessions respectively from the downloaded GO annotation file, used when ImportDirectly is set to True to obtain a new GO association file

GOURL: str, Default "http://current.geneontology.org/annotations/" The location (base URL) where the GO association annotation files are downloaded from

Returns:

dictionary Dictionary of IDToGO and GOToID dictionaries

Usage: GOassignment = GOAnalysisAssigner()

obtainConstantGeneDictionary(*GeneDictionary*, *GetGeneDictionaryOptions*, *AugmentDictionary*)

Obtain gene dictionary - if it exists can either augment with new information or Species or create new, if not exist then create variable.

Parameters:

GeneDictionary: dictionary or None An existing variable to use as a gene dictionary in annotations. If set to None the default ConstantGeneDictionary will be used

GetGeneDictionaryOptions: dictionary A list of options that will be passed to this internal GetGeneDictionary function

AugmentDictionary: boolean A choice whether or not to augment the current ConstantGeneDictionary global variable or create a new one

Returns: None

Usage: obtainConstantGeneDictionary(None, {}, False)

GOAnalysis(*data, GetGeneDictionaryOptions={}, AugmentDictionary=True, InputID=['UniProt ID', 'Gene Symbol'], OutputID='UniProt ID', GOAnalysisAssignerOptions={}, BackgroundSet=[], Species='human', OntologyLengthFilter=2, ReportFilter=1, ReportFilterFunction=<ufunc 'greater_equal'>, pValueCutoff=0.05, TestFunction=<function <lambda>>, HypothesisFunction=<function <lambda>>, FilterSignificant=True, OBODictionaryVariable=None, OBOGODictionaryOptions={}, MultipleListCorrection=None, MultipleList=False, GeneDictionary=None*)

Calculate input data over-representation analysis for Gene Ontology (GO) categories.

Parameters:

data: pd.DataFrame or list Data to analyze

GetGeneDictionaryOptions: dictionary, Default {} A list of options that will be passed to this internal GetGeneDictionary function

AugmentDictionary: boolean, Default True A choice whether or not to augment the current ConstantGeneDictionary global variable or create a new one

InputID: list, Default ["UniProt ID","Gene Symbol"] Kind of identifiers/accessions used as input

OutputID: str, Default "UniProt ID" Kind of IDs/accessions to convert the input IDs/accession numbers in the function's analysis

GOAnalysisAssignerOptions: dictionary, Default {} A list of options that will be passed to the internal GOAnalysisAssigner function

BackgroundSet: list, Default [] Background list to create annotation projection to limited background space, involves considering pathways/groups/sets and that provides a list of IDs (e.g. gene accessions) that should be considered as the background for the calculation

Species: str, Default "human" The species considered in the calculation, by default corresponding to human

OntologyLengthFilter: int, Default 2 Function that can be used to set the value for which terms to consider in the computation, by excluding GO terms that have fewer items compared to the OntologyLengthFilter value. It is used by the internal GOAnalysisAssigner function

ReportFilter: int, Default 1 Functions that use pathways/ontologies/groups, and provides a cutoff for membership in ontologies/pathways/groups in selecting which terms/categories to return. It is typically used in conjunction with ReportFilterFunction

ReportFilterFunction: function , Default np.greater_equal Specifies what operator form will be used to compare against ReportFilter option value in selecting which terms/categories to return

pValueCutoff: float, Default 0.05 Significance cutoff

TestFunction: function, Default lambda n, N, M, x: 1. - scipy.stats.hypergeom.cdf(x-1, M, n, N)
Test function

HypothesisFunction: function, Default lambda data, SignificanceLevel: BenjaminiHochbergFDR(data, Significance[illegible]
Allows the choice of function for implementing multiple hypothesis testing considerations

FilterSignificant: boolean, Default True Can be set to True to filter data based on whether the analysis result is statistically significant, or if set to False to return all membership computations

OBODictionaryVariable: str, Default None A GO annotation variable. If set to None, OBOGODictionary will be used internally to automatically generate the default GO annotation

OBOGODictionaryOptions: dictionary, Default {} A list of options to be passed to the internal OBOGODictionary function that provides the GO annotations

MultipleListCorrection: boolean, Default None Specifies whether or not to correct for multi-omics analysis. The choices are None, Automatic, or a custom number, e.g protein+RNA

MultipleList: boolean, Default False Specifies whether the input accessions list constituted a multi-omics list input that is annotated so

GeneDictionary: str, Default None Points to an existing variable to use as a gene dictionary in annotations. If set to None the default ConstantGeneDictionary will be used

Returns:

dictionary Enrichment dictionary

Usage:

goExample1 = GOAnalysis([“TAB1”, “TNFSF13B”, “MALT1”, “TIRAP”, “CHUK”, “TNFRSF13C”, “PARP1”, “CSNK2A1”, “CSNK2A2”, “CSNK2B”, “LTBR”, “LYN”, “MYD88”, “GADD45B”, “ATM”, “NFKB1”, “NFKB2”, “NFKBIA”, “IRAK4”, “PIAS4”, “PLAU”])

GeneTranslation (*InputList*, *TargetIDList*, *GeneDictionary*, *InputID=None*, *Species='human'*)
Use geneDictionary to convert inputList IDs to different annotations as indicated by targetIDList.

Parameters:

InputList: list List of names

TargetIDList: list Target ID list

GeneDictionary: dictionary An existing variable to use as a gene dictionary in annotations. If set to None the default ConstantGeneDictionary will be used

InputID: str, Default None The kind of identifiers/accessions used as input

Species: str, Default “human” The species considered in the calculation, by default corresponding to human

Returns:

dictionary Gene dictionary

Usage: GenDict = GeneTranslation(data, “UniProt ID”, ConstantGeneDictionary, InputID = [“UniProt ID”,”Gene Symbol”], Species = “human”)

KEGGAnalysisAssigner (*PyIOmicaDataDirectory=None*, *ImportDirectly=False*, *BackgroundSet=[]*, *KEGGQuery1='pathway'*, *KEGGQuery2='hsa'*, *LengthFilter=None*, *LengthFilterFunction=<ufunc 'greater_equal'>*, *Labels=['IDToPath', 'PathToID']*)
Create KEGG: Kyoto Encyclopedia of Genes and Genomes pathway associations, restricted to required background set, downloading the data if necessary.

Parameters:

PyIOmicaDataDirectory: str, Default None Directory where the default package data is stored

ImportDirectly: boolean, Default False Import from URL regardles is the file already exists

BackgroundSet: list, Default [] A list of IDs (e.g. gene accessions) that should be considered as the background for the calculation

KEGGQuery1: str, Default "pathway" Make KEGG API calls, and sets string query1 in http://rest.kegg.jp/link/<> query1 <> / <> query2. Typically this will be used as the target database to find related entries by using database cross-references

KEGGQuery2: str, Default "hsa" KEGG API calls, and sets string query2 in http://rest.kegg.jp/link/<> query1 <> / <> query2. Typically this will be used as the source database to find related entries by using database cross-references

LengthFilterFunction: function, Default np.greater_equal Option for functions that perform computations of membership in pathways/ontologies/groups/sets, that specifies which function to use to filter the number of members a reported category has compared to the number typically provided by LengthFilter

LengthFilter: int, Default None Allows the selection of how many members each category can have, as typically restricted by the LengthFilterFunction

Labels: list, Default ["IDToPath", "PathToID"] A string list for how keys in a created association will be named

Returns:

dictionary IDToPath and PathToID dictionary

Usage: KEGGassignment = KEGGAnalysisAssigner()

KEGGDictionary(*PyIOmicaDataDirectory=None, ImportDirectly=False, KEGGQuery1='pathway', KEGGQuery2='hsa'*)

Create a dictionary from KEGG: Kyoto Encyclopedia of Genes and Genomes terms - typically association of pathways and members therein.

Parameters:

PyIOmicaDataDirectory: str, Default None directory where the default package data is stored

ImportDirectly: boolean, Default False import from URL regardles is the file already exists

KEGGQuery1: str, Default "pathway" make KEGG API calls, and sets string query1 in http://rest.kegg.jp/link/<> query1 <> / <> query2. Typically this will be used as the target database to find related entries by using database cross-references

KEGGQuery2: str, Default "hsa" KEGG API calls, and sets string query2 in http://rest.kegg.jp/link/<> query1 <> / <> query2. Typically this will be used as the source database to find related entries by using database cross-references

Returns:

dictionary Dictionary of definitions

Usage: KEGGDict = KEGGDictionary()

KEGGAnalysis(*data, AnalysisType='Genomic', GetGeneDictionaryOptions={}, AugmentDictionary=True, InputID=['UniProt ID', 'Gene Symbol'], OutputID='KEGG Gene ID', MolecularInputID=['cpd'], MolecularOutputID='cpd', KEGGAnalysisAssignerOptions={}, BackgroundSet=[], KEGGOrganism='hsa', KEGGMolecular='cpd', KEGGDatabase='pathway', PathwayLengthFilter=2, ReportFilter=1, ReportFilterFunction=<ufunc 'greater_equal'>, pValueCutoff=0.05, TestFunction=<function <lambda>>, HypothesisFunction=<function <lambda>>, FilterSignificant=True, KEGGDictionaryVariable=None, KEGGDictionaryOptions={}, MultipleListCorrection=None, MultipleList=False, GeneDictionary=None, Species='human', MolecularSpecies='compound', NonUCSC=False, PyIOmicaDataDirectory=None*)

Calculate input data over-representation analysis for KEGG: Kyoto Encyclopedia of Genes and Genomes pathways. Input can be a list, a dictionary of lists or a clustering object.

Parameters:

data: pandas.DetaFrame or list Data to analyze

AnalysisType: str, Default "Genomic" Analysis methods that may be used, "Genomic", "Molecular" or "All"

GetGeneDictionaryOptions: dictionary, Default {} A list of options that will be passed to this internal GetGeneDictionary function

AugmentDictionary: boolean, Default True A choice whether or not to augment the current ConstantGeneDictionary global variable or create a new one

InputID: list, Default ["UniProt ID", "Gene Symbol"] The kind of identifiers/accessions used as input

OutputID: str, Default "KEGG Gene ID" A string value that specifies what kind of IDs/accessions to convert the input IDs/accession numbers in the function's analysis

MolecularInputID: list, Default ["cpd"] A string list to indicate the kind of ID to use for the input molecule entries

MolecularOutputID: str, Default "cpd" A string list to indicate the kind of ID to use for the input molecule entries

KEGGAnalysisAssignerOptions: dictionary, Default {} A list of options that will be passed to this internal KEGGAnalysisAssigner function

BackgroundSet: list, Default [] A list of IDs (e.g. gene accessions) that should be considered as the background for the calculation

KEGGOrganism: str, Default "hsa" Indicates which organism (org) to use for "Genomic" type of analysis (default is human analysis: org="hsa")

KEGGMolecular: str, Default "cpd" Which database to use for molecular analysis (default is the compound database: cpd)

KEGGDatabase: str, Default "pathway" KEGG database to use as the target database

PathwayLengthFilter: int, Default 2 Pathways to consider in the computation, by excluding pathways that have fewer items compared to the PathwayLengthFilter value

ReportFilter: int, Default 1 Provides a cutoff for membership in ontologies/pathways/groups in selecting which terms/categories to return. It is typically used in conjunction with ReportFilterFunction

ReportFilterFunction: function, Default np.greater_equal Operator form will be used to compare against ReportFilter option value in selecting which terms/categories to return

pValueCutoff: float, Default 0.05 A cutoff p-value for (adjusted) p-values to assess statistical significance

TestFunction: function, Default lambda n, N, M, x: 1. - scipy.stats.hypergeom.cdf(x-1, M, n, N) A function used to calculate p-values

HypothesisFunction: function, Default lambda data, SignificanceLevel: BenjaminiHochbergFDR(data, SignificanceI
Allows the choice of function for implementing multiple hypothesis testing considerations

FilterSignificant: boolean, Default True Can be set to True to filter data based on whether the analysis result is statistically significant, or if set to False to return all membership computations

KEGGDictionaryVariable: str, Default None KEGG dictionary, and provides a KEGG annotation variable. If set to None, KEGGDictionary will be used internally to automatically generate the default KEGG annotation

KEGGDictionaryOptions: dictionary, Default {} A list of options to be passed to the internal KEGGDictionary function that provides the KEGG annotations

MultipleListCorrection: boolean, Default None Specifies whether or not to correct for multi-omics analysis. The choices are None, Automatic, or a custom number

MultipleList: boolean, Default False Whether the input accessions list constituted a multi-omics list input that is annotated so

GeneDictionary: str, Default None Existing variable to use as a gene dictionary in annotations. If set to None the default ConstantGeneDictionary will be used

Species: str, Default "human" The species considered in the calculation, by default corresponding to human

MolecularSpecies: str, Default "compound" The kind of molecular input

NonUCSC: , Default If UCSC browser was used in determining an internal GeneDictionary used in ID translations, where the KEGG identifiers for genes are number strings (e.g. 4790).The NonUCSC option can be set to True if standard KEGG accessions are used in a user provided GeneDictionary variable, in the form OptionValue[KEGGOrganism] <>:<>numberString, e.g. hsa:4790

PyIOmicaDataDirectory: str, Default None Directory where the default package data is stored

Returns:

dictionary Enrichment dictionary

Usage:

keggExample1 = KEGGAnalysis(["TAB1", "TNFSF13B", "MALT1", "TIRAP", "CHUK", "TNFRSF13C", "PARP1 "GADD45B", "ATM", "NFKB1", "NFKB2", "NFKBIA", "IRAK4", "PIAS4", "PLAU", "POLR3B", "NME1", "CTPS1", "POLR3A"])

MassMatcher (*data*, *accuracy*, *MassDictionaryVariable=None*, *MolecularSpecies='cpd'*)

Assign putative mass identification to input data based on monoisotopic mass (using PyIOmica's mass dictionary). The accuracy in parts per million.

Parameters:

data: np.array Input data

accuracy: float Accuracy

MassDictionaryVariable: boolean, Default None Mass dictionary variable. If set to None, inbuilt mass dictionary (MassDictionary) will be loaded and used

MolecularSpecies: str, Default "cpd" The kind of molecular input

Returns:

list List of IDs

Usage: result = MassMatcher(18.010565, 2)

MassDictionary (*PyIOmicaDataDirectory=None*)

Load PyIOmica's current mass dictionary.

Parameters:

PyIOmicaDataDirectory: str, Default None Directory where the default package data is stored

Returns:

dictionary Mass dictionary

Usage: MassDict = MassDictionary()

ExportEnrichmentReport (*data*, *AppendString="*, *OutputDirectory=None*)

Export results from enrichment analysis to Excel spreadsheets.

Parameters:

data: dictionary Enrichment results

AppendString: str, Default "" Custom report name, if empty then time stamp will be used

OutputDirectory: boolean, Default None Path of directories where the report will be saved

Returns: None

Usage: ExportEnrichmentReport(goExample1, AppendString='goExample1', OutputDirectory=None)

BenjaminiHochbergFDR(*pValues*, *SignificanceLevel=0.05*)
HypothesisTesting BenjaminiHochbergFDR correction

Parameters:

pValues: 1d numpy.array Array of p-values

SignificanceLevel: float, Default 0.05 Significance level

Returns:

dictionary Corrected p-Values, p- and q-Value cuttoffs

Usage: result = BenjaminiHochbergFDR(pValues)

3.4 Extended DataFrame and data-processing functions

Submodule **pyiomica.extendedDataFrame**

PyIOmica Dataframe extending Pandas DataFrame with new functions

Classes

DataFrame([data, index, columns, dtype, copy])	Class based on pandas.DataFrame extending capabilities into the doamin of PyIOmica

Functions

getLobmScarglePeriodogramOfDataframe(df_data)	Calculate Lobm-Scargle periodogram of DataFrame.
getRandomAutocorrelations(df_data[, ...])	Generate autocorrelation null-distribution from permutated data using Lomb-Scargle Autocorrelation.
getRandomPeriodograms(df_data[, ...])	Generate periodograms null-distribution from permutated data using Lomb-Scargle function.
getRandomSpikesCutoffs(df_data, p_cutoff[, ...])	Calculate spikes cuttoffs from a bootstrap of provided data, gived the significance cutoff p_cutoff.
mergeDataframes(listOfDataframes[, axis])	Merge a list of Dataframes (outer join).

class DataFrame(*data=None*, *index=None*, *columns=None*, *dtype=None*, *copy=False*)
Bases: `pandas.core.frame.DataFrame`

Class based on pandas.DataFrame extending capabilities into the doamin of PyIOmica

Initialization parameters are identical to those in pandas.DataFrame See https://pandas.pydata.org/pandas-docs/stable/reference/api/pandas.DataFrame.html for detail. **Methods**

`__init__`([data, index, columns, dtype, copy])	Initialization method
`boxCoxTransform`([axis, inplace])	Box-cox transform data.
`compareTimeSeriesToPoint`([point, inplace])	Subtract a particular point of each time series (row) of a Dataframe.
`compareTwoTimeSeries`(df[, function, …])	Create a new Dataframe based on comparison of two existing Dataframes.
`filterOutAllZeroSignals`([inplace])	Filter out all-zero signals from a DataFrame.
`filterOutFractionMissingSignals`(…[, inplace])	Filter out fraction-zero signals from a DataFrame.
`filterOutFractionZeroSignals`(…[, inplace])	Filter out fraction-zero signals from a DataFrame.
`filterOutReferencePointZeroSignals`([…])	Filter out out first time point zeros signals from a DataFrame.
`imputeMissingWithMedian`([axis, inplace])	Normalize signals to unity.
`modifiedZScore`([axis, inplace])	Z-score (Median-based) transform data.
`normalizeSignalsToUnity`([referencePoint, …])	Normalize signals to unity.
`quantileNormalize`([output_distribution, …])	Quantile Normalize signals in a DataFrame.
`removeConstantSignals`(theta_cutoff[, inplace])	Remove constant signals.
`tagLowValues`(cutoff, replacement[, inplace])	Tag low values with replacement value.
`tagMissingAsValue`([value, inplace])	Tag NaN with zero.
`tagValueAsMissing`([value, inplace])	Tag zero values with NaN.

`__init__`(*data=None*, *index=None*, *columns=None*, *dtype=None*, *copy=False*)

Initialization method

`filterOutAllZeroSignals`(*inplace=False*)

Filter out all-zero signals from a DataFrame.

Parameters:

inplace: boolean, Default False Whether to modify data in place or return a new one

Returns:

Dataframe or None Processed data

Usage: df_data = df_data.filterOutAllZeroSignals()

or

df_data.filterOutAllZeroSignalse(inplace=True)

`filterOutFractionZeroSignals`(*min_fraction_of_non_zeros*, *inplace=False*)

Filter out fraction-zero signals from a DataFrame.

Parameters:

min_fraction_of_non_zeros: float Maximum fraction of allowed zeros

inplace: boolean, Default False Whether to modify data in place or return a new one

Returns:

Dataframe or None Processed data

Usage: df_data = df_data.filterOutFractionZeroSignals(0.75)

or

df_data.filterOutFractionZeroSignals(0.75, inplace=True)

filterOutFractionMissingSignals(*min_fraction_of_non_missing*, *inplace=False*)
Filter out fraction-zero signals from a DataFrame.

Parameters:

min_fraction_of_non_missing: float Maximum fraction of allowed zeros

inplace: boolean, Default False Whether to modify data in place or return a new one

Returns:

Dataframe or None Processed data

Usage: df_data = df_data.filterOutFractionMissingSignals(0.75)

or

df_data.filterOutFractionMissingSignals(0.75, inplace=True)

filterOutReferencePointZeroSignals(*referencePoint=0*, *inplace=False*)
Filter out out first time point zeros signals from a DataFrame.

Parameters:

referencePoint: int, Default 0 Index of the reference point

inplace: boolean, Default False Whether to modify data in place or return a new one

Returns:

Dataframe or None Processed data

Usage: df_data = df_data.filterOutFirstPointZeroSignals()

or

df_data.filterOutFirstPointZeroSignals(inplace=True)

tagValueAsMissing(*value=0.0*, *inplace=False*)
Tag zero values with NaN.

Parameters:

inplace: boolean, Default False Whether to modify data in place or return a new one

Returns:

Dataframe or None Processed data

Usage: df_data = df_data.tagValueAsMissing()

or

df_data.tagValueAsMissing(inplace=True)

tagMissingAsValue(*value=0.0*, *inplace=False*)
Tag NaN with zero.

Parameters:

inplace: boolean, Default False Whether to modify data in place or return a new one

Returns:

Dataframe or None Processed data

Usage: df_data = df_data.tagMissingAsValue()

or

df_data.tagMissingAsValue(inplace=True)

tagLowValues(*cutoff*, *replacement*, *inplace=False*)
Tag low values with replacement value.

Parameters:

cutoff: float Values below the "cutoff" are replaced with "replacement" value

replacement: float Values below the "cutoff" are replaced with "replacement" value

inplace: boolean, Default False Whether to modify data in place or return a new one

Returns:

Dataframe or None Processed data

Usage: df_data = df_data.tagLowValues(1., 1.)

or

df_data.tagLowValues(1., 1., inplace=True)

removeConstantSignals(*theta_cutoff*, *inplace=False*)
Remove constant signals.

Parameters:

theta_cutoff: float Parameter for filtering the signals

inplace: boolean, Default False Whether to modify data in place or return a new one

Returns:

Dataframe or None Processed data

Usage: df_data = df_data.removeConstantSignals(0.3)

or

df_data.removeConstantSignals(0.3, inplace=True)

boxCoxTransform(*axis=1*, *inplace=False*)
Box-cox transform data.

Parameters:

axis: int, Default 1 Direction of processing, columns (1) or rows (0)

inplace: boolean, Default False Whether to modify data in place or return a new one

Returns:

Dataframe or None Processed data

Usage: df_data = df_data.boxCoxTransformDataframe()

or

df_data.boxCoxTransformDataframe(inplace=True)

modifiedZScore(*axis=0*, *inplace=False*)
Z-score (Median-based) transform data.

Parameters:

axis: int, Default 1 Direction of processing, rows (1) or columns (0)

inplace: boolean, Default False Whether to modify data in place or return a new one

Returns:

Dataframe or None Processed data

Usage: df_data = df_data.modifiedZScoreDataframe()

or

df_data.modifiedZScoreDataframe(inplace=True)

`normalizeSignalsToUnity`(*referencePoint=0*, *inplace=False*)

Normalize signals to unity.

Parameters:

referencePoint: int, Default 0 Index of the reference point

inplace: boolean, Default False Whether to modify data in place or return a new one

Returns:

Dataframe or None Processed data

Usage: df_data = df_data.normalizeSignalsToUnityDataframe()

or

df_data.normalizeSignalsToUnityDataframe(inplace=True)

`quantileNormalize`(*output_distribution='original'*, *averaging=<function mean>*, *ties=<function mean>*, *inplace=False*)

Quantile Normalize signals in a DataFrame.

Note that it is possible there may be equal values within the dataset. In such a scenario, by default, the quantile normalization implementation considered here works by replacing the degenerate values with the mean over all the degenerate ranks. Note, that for the default option to work the data should not have any missing values. If output_distribution is set to 'uniform' or 'normal' then the scikit-learn's Quantile Transformation is used.

Parameters:

output_distribution: str, Default 'original' Output distribution. Other options are 'normal' and 'uniform'

averaging: function, Default np.mean With what value to replace the same-rank elements across samples. Default is to take the mean of same-rank elements

ties: function or str, Default np.mean Function or name of the function. How ties should be handled. Default is to replace ties with their mean. Other possible options are: 'mean', 'median', 'prod', 'sum', etc.

inplace: boolean, Default False Whether to modify data in place or return a new one

Returns:

Dataframe or None Processed data

Usage: df_data = pd.DataFrame(index=['Gene 1','Gene 2','Gene 3','Gene 4'], columns=['Col 0','Col 1','Col 2'], data=np.array([[5, 4, 3], [2, 1, 4], [3, 4, 6], [4, 2, 8]]))

df_data = df_data.quantileNormalize()

or

df_data.df_data.quantileNormalize(inplace=True)

compareTimeSeriesToPoint (*point='first'*, *inplace=False*)
Subtract a particular point of each time series (row) of a Dataframe.

Parameters:

point: str, int or float Possible options are 'first', 'last', 0, 1, ... , 10, or a value.

inplace: boolean, Default False Whether to modify data in place or return a new one

Returns:

Dataframe or None Processed data

Usage: df_data = df_data.compareTimeSeriesToPoint()

or

df_data.compareTimeSeriesToPoint(df_data)

compareTwoTimeSeries (*df*, *function=<ufunc 'subtract'>*, *compareAllLevelsInIndex=True*, *mergeFunction=<function mean>*)
Create a new Dataframe based on comparison of two existing Dataframes.

Parameters:

df: pandas.DataFrame Data to compare

function: function, Default np.subtract Other options are np.add, np.divide, or another <ufunc>.

compareAllLevelsInIndex: boolean, Default True Whether to compare all levels in index. If False only "source" and "id" will be compared

mergeFunction: function, Default np.mean Input Dataframes are merged with this function, i.e. np.mean (default), np.median, np.max, or another <ufunc>.

Returns:

DataFrame or None Processed data

Usage: df_data = compareTwoTimeSeries(df_dataH2, df_dataH1, function=np.subtract, compareAllLevelsInIndex=False, mergeFunction=np.median)

imputeMissingWithMedian (*axis=1*, *inplace=False*)
Normalize signals to unity.

Parameters:

axis: int, Default 1 Axis to apply trasnformation along

inplace: boolean, Default False Whether to modify data in place or return a new one

Returns:

Dataframe or None Processed data

Usage: df_data = df_data.imputeMissingWithMedian()

or

df_data.imputeMissingWithMedian(inplace=True)

mergeDataframes (*listOfDataframes*, *axis=0*)
Merge a list of Dataframes (outer join).

Parameters:

listOfDataframes: list List of pandas.DataFrames

axis: int, Default 0 Merge direction. 0 to stack vertically, 1 to stack horizontally

Returns:

pandas.Dataframe Processed data

Usage: df_data = mergeDataframes([df_data1, df_data2])

getLobmScarglePeriodogramOfDataframe (*df_data*, *NumberOfCPUs=4*, *parallel=True*)
Calculate Lobm-Scargle periodogram of DataFrame.

Parameters:

df: pandas.DataFrame Data to process

parallel: boolean, Default True Whether to calculate in parallel mode (>1 process)

NumberOfCPUs: int, Default 4 Number of processes to create if parallel is True

Returns:

pandas.Dataframe Lomb-Scargle periodograms

Usage: df_periodograms = getLobmScarglePeriodogramOfDataframe(df_data)

getRandomSpikesCutoffs (*df_data*, *p_cutoff*, *NumberOfRandomSamples=1000*)
Calculate spikes cuttoffs from a bootstrap of provided data, gived the significance cutoff p_cutoff.

Parameters:

df_data: pandas.DataFrame Data where rows are normalized signals

p_cutoff: float p-Value cutoff, e.g. 0.01

NumberOfRandomSamples: int, Default 1000 Size of the bootstrap distribution

Returns:

dictionary Dictionary of spike cutoffs.

Usage: cutoffs = getSpikesCutoffs(df_data, 0.01)

getRandomAutocorrelations (*df_data*, *NumberOfRandomSamples=100000*, *NumberOfCPUs=4*, *fraction=0.75*, *referencePoint=0*)
Generate autocorrelation null-distribution from permutated data using Lomb-Scargle Autocorrelation. NOTE: there should be already no missing or non-numeric points in the input Series or Dataframe

Parameters: df_data: pandas.Series or pandas.Dataframe

NumberOfRandomSamples: int, Default 105** Size of the distribution to generate

NumberOfCPUs: int, Default 4 Number of processes to run simultaneously

Returns:

pandas.DataFrame Dataframe containing autocorrelations of null-distribution of data.

Usage: result = getRandomAutocorrelations(df_data)

getRandomPeriodograms (*df_data*, *NumberOfRandomSamples=100000*, *NumberOfCPUs=4*, *fraction=0.75*, *referencePoint=0*)
Generate periodograms null-distribution from permutated data using Lomb-Scargle function.

Parameters: df_data: pandas.Series or pandas.Dataframe

NumberOfRandomSamples: int, Default 105** Size of the distribution to generate

NumberOfCPUs: int, Default 4 Number of processes to run simultaneously

Returns:

pandas.DataFrame Dataframe containing periodograms

Usage: result = getRandomPeriodograms(df_data)

3.5 Clustering functions

Submodule **pyiomica.clusteringFunctions**

Clustering-related functions

Functions

`exportClusteringObject`(ClusteringObject, . . .)	Export a clustering Groups-Subgroups dictionary object to a SpreadSheet.
`getCommunitiesOfTimeSeries`(data, times[, . . .])	Get communities of time series
`getEstimatedNumberOfClusters`(data, . . . [, . . .])	Get estimated number of clusters using ARI with KMeans
`getGroupingIndex`(data[, n_groups, method, . . .])	Cluster data into N groups, if N is provided, else determine N return: linkage matrix, cluster labels, possible cluster labels.
`get_n_clusters_from_linkage_Elbow`(Y)	Get optimal number clusters from linkage.
`get_n_clusters_from_linkage_Silhouette`(Y, . . .)	Determine the optimal number of cluster in data maximizing the Silhouette score.
`makeClusteringObject`(df_data, df_data_autocorr)	Make a clustering Groups-Subgroups dictionary object.
`runForClusterNum`(arguments)	Calculate Adjusted Rand Index of the data for a range of cluster numbers.

getEstimatedNumberOfClusters(*data*, *cluster_num_min*, *cluster_num_max*, *trials_to_do*, *numberOfAvailableCPUs=4*, *plotID=None*, *printScores=False*)

Get estimated number of clusters using ARI with KMeans

Parameters:

data: 2d numpy.array Data to analyze

cluster_num_min: int Minimum possible number of clusters

cluster_num_max: int Maximum possible number of clusters

trials_to_do: int Number of trials to do in ARI function

numberOfAvailableCPUs: int, Default 4 Number of processes to run in parallel

plotID: str, Default None Label for the plot of peaks

printScores: boolean, Default False Whether to print all scores

Returns:

tuple Largest peak, other possible peaks.

Usage: n_clusters = getEstimatedNumberOfClusters(data, 1, 20, 25)

get_n_clusters_from_linkage_Elbow(*Y*)

Get optimal number clusters from linkage. A point of the highest accelleration of the fusion coefficient of the given linkage.

Parameters:

Y: 2d numpy.array Linkage matrix

Returns:

int Optimal number of clusters

Usage: n_clusters = get_n_clusters_from_linkage_Elbow(Y)

`get_n_clusters_from_linkage_Silhouette` (*Y*, *data*, *metric*)

Determine the optimal number of cluster in data maximizing the Silhouette score.

Parameters:

Y: 2d numpy.array Linkage matrix

data: 2d numpy.array Data to analyze

metric: str or function Distance measure

Returns:

int Optimal number of clusters

Usage: n_clusters = get_n_clusters_from_linkage_Silhouette(Y, data, 'euclidean')

`runForClusterNum` (*arguments*)

Calculate Adjusted Rand Index of the data for a range of cluster numbers.

Parameters:

arguments: tuple

A tuple of three parameters int the form (cluster_num, data_array, trials_to_do), where

cluster_num: int Maximum number of clusters

data_array: 2d numpy.array Data to test

trials_to_do: int Number of trials for each cluster number

Returns:

1d numpy.array Numpy array

Usage: instPool = multiprocessing.Pool(processes = NumberOfAvailableCPUs)

scores = instPool.map(runForClusterNum, [(cluster_num, copy.deepcopy(data), trials_to_do) for cluster_num in range(cluster_num_min, cluster_num_max + 1)])

instPool.close()

instPool.join()

`getGroupingIndex` (*data*, *n_groups=None*, *method='weighted'*, *metric='correlation'*, *significance='Elbow'*)

Cluster data into N groups, if N is provided, else determine N return: linkage matrix, cluster labels, possible cluster labels.

Parameters:

data: 2d numpy.array Data to analyze

n_groups: int, Default None Number of groups to split data into

method: str, Default 'weighted' Linkage calculation method

metric: str, Default 'correlation' Distance measure

significance: str, Default 'Elbow' Method for determining optimal number of groups and subgroups

Returns:

tuple Linkage matrix, cluster index, possible groups

Usage: x, y, z = getGroupingIndex(data, method='weighted', metric='correlation', significance='Elbow')

makeClusteringObject(*df_data*, *df_data_autocorr*, *method='weighted'*, *metric='correlation'*, *significance='Elbow'*)

Make a clustering Groups-Subgroups dictionary object.

Parameters:

df_data: pandas.DataFrame Data to analyze in DataFrame format

df_data_autocorr: pandas.DataFrame Autocorrelations or periodograms in DataFrame format

method: str, Default 'weighted' Linkage calculation method

metric: str, Default 'correlation' Distance measure

significance: str, Default 'Elbow' Method for determining optimal number of groups and subgroups

Returns:

dictionary Clustering object

Usage: myObj = makeClusteringObject(df_data, df_data_autocorr, significance='Elbow')

exportClusteringObject(*ClusteringObject*, *saveDir*, *dataName*, *includeData=True*, *includeAutocorr=True*)

Export a clustering Groups-Subgroups dictionary object to a SpreadSheet. Linkage data is not exported.

Parameters:

ClusteringObject: dictionary Clustering object

saveDir: str Path of directories to save the object to

dataName: str Label to include in the file name

includeData: boolean, Default True Export data

includeAutocorr: boolean, Default True Export autocorrelations of data

Returns:

str File name of the exported clustering object

Usage: exportClusteringObject(myObj, '/dir1', 'myObj')

getCommunitiesOfTimeSeries(*data*, *times*, *numberOfCommunities=1*, *horizontal=False*, *method='betweenness_centrality'*)

Get communities of time series

Parameters:

data: 1d numpy.array Data array

times: 1d numpy.array Times corresponding to data points

numberOfCommunities: int, Default 1 Number of communities to find

horizontal: boolean, Default False Whether to use horizontal or normal visibility graph

method: str, Default 'betweenness_centrality' Name of the method to use

Returns:

(list, graph) List of communities and a networkx graph

Usage: getCommunitiesOfTimeSeries(data, times, numberOfCommunities=5)

3.6 Visibility graph preparation functions

Submodule **pyiomica.visibilityGraphAuxiliaryFunctions**

Functions to generate adjacency matrix of visibility graphs

Miscellaneous

getAdjacencyMatrixOfHVG(*args, **kwargs)	Calculate adjacency matrix of horizontal visibility graph.
getAdjacencyMatrixOfNVG(*args, **kwargs)	Calculate adjacency matrix of visibility graph.

Functions

getAdjacencyMatrixOfHVGbyNUMPY(data)	Calculate adjacency matrix of horizontal visibility graph.
getAdjacencyMatrixOfNVGbyNUMPY(data, times)	Calculate adjacency matrix of visibility graph.

getAdjacencyMatrixOfNVG(**args*, ***kwargs*)

Calculate adjacency matrix of visibility graph. JIT-accelerated version (a bit faster than NumPy-accelerated version). Allows use of Multiple CPUs.

Parameters:

data: 2d numpy.array Numpy array of floats

times: 1d numpy.array Numpy array of floats

Returns:

2d numpy.array Adjacency matrix

Usage: A = getAdjacencyMatrixOfNVG(data, times)

getAdjacencyMatrixOfNVGbyNUMPY(*data*, *times*)

Calculate adjacency matrix of visibility graph. NumPy-accelerated version. Somewhat slower than JIT-accelerated version. Use in serial applications.

Parameters:

data: 2d numpy.array Numpy array of floats

times: 1d numpy.array Numpy array of floats

Returns:

2d numpy.array Adjacency matrix

Usage: A = getAdjacencyMatrixOfNVGbyNUMPY(data, times)

getAdjacencyMatrixOfHVG(**args*, ***kwargs*)

Calculate adjacency matrix of horizontal visibility graph. JIT-accelerated version (a bit faster than NumPy-accelerated version). Single-threaded beats NumPy up to 2k data sizes. Allows use of Multiple CPUs.

Parameters:

data: 2d numpy.array Numpy array of floats

Returns:

2d numpy.array Adjacency matrix

Usage: A = getAdjacencyMatrixOfHVG(data)

getAdjacencyMatrixOfHVGbyNUMPY (*data*)

Calculate adjacency matrix of horizontal visibility graph. NumPy-accelerated version. Use with datasets larger than 2k. Use in serial applications.

Parameters:

data: 2d numpy.array Numpy array of floats

Returns:

2d numpy.array Adjacency matrix

Usage: A = getAdjacencyMatrixOfHVGbyNUMPY(data)

3.7 Visualization functions

Submodule **pyiomica.visualizationFunctions**

Visualization functions

Functions

addColorbarToFigure(fig, data[, ...])	Add colorbar to figure
addVisibilityGraph(data, times[, dataName, ...])	Draw a Visibility graph of data on a provided Matplotlib figure.
makeDataHistograms(df, saveDir, dataName[, ...])	Make a histogram for each Series (time point) in a Dataframe.
makeDendrogramHeatmapOfClusteringObject(...)	Make Dendrogram-Heatmap plot along with VIsibility graphs.
makeLombScarglePeriodograms(df, saveDir, ...)	Make a combined plot of the signal and its Lomb-Scargle periodogram for each pandas Series (time point) in a Dataframe.
makePlotOfPeak(data_all, scores, ...)	Plot peaks.
makeVisibilityBarGraph(data, times, saveDir, ...)	Bar-plot style visibility graph.
makeVisibilityGraph(intensities, positions, ...)	Make either horizonral or normal visibility graph of a time series using function addVisibilityGraph.
saveFigure(fig, saveDir, label, extension, dpi)	Function primarily used internally to save and close figures

saveFigure (*fig*, *saveDir*, *label*, *extension*, *dpi*, *close=True*)

Function primarily used internally to save and close figures

Parameters:

saveDir: str Path of directories to save the object to

extension: str, Default '.png' Path of directories to save the object to

dpi: int, Default 300 Figure resolution if rasterized

close: boolean: Default True Whether to close the figure after saving

Returns: None

Usage: saveFigure(fig, saveDir, label, extension, dpi)

makeDataHistograms(*df, saveDir, dataName, figsize=(8, 8), range_min=<function amin>, range_max=<function amax>, includeTitle=True, title='Data @ timePoint:', fontsize=8, fontcolor='b', N_bins=100, color='b', extension='.png', dpi=300*)

Make a histogram for each Series (time point) in a Dataframe.

Parameters:

df: pandas.DataFrame Data to visualize

saveDir: str Path of directories to save the object to

dataName: str Label to include in the file name

figsize: tuple, Default (8,8) Size of the figure in inches

range_min: str, Default How to determine data minimum

range_max: int, float or function, Default How to determine data maximum

includeTitle: boolean, Default True Path of directories to save the object to

title: str, Default 'Data @ timePoint:' Text of the title

fontsize: str, Default 8 Fontsize of the labels

fontcolor: str, Default 'b' Color of the title font

N_bins: int, Default 100 Number of bins in the histogram

color: str, Default 'b' Color of the bars

extension: str, Default '.png' Path of directories to save the object to

dpi: int, Default 300 Figure resolution if rasterized

Returns: None

Usage: makeDataHistograms(df, '/dir1', 'myData')

makeLombScarglePeriodograms(*df, saveDir, dataName, minNumberOfNonzeroPoints=5, oversamplingRate=100, figsize=(5, 5), title1='TimeSeries Data', title2='Lomb-Scargle periodogram', extension='.png', dpi=300*)

Make a combined plot of the signal and its Lomb-Scargle periodogram for each pandas Series (time point) in a Dataframe.

Parameters:

df: pandas.DataFrame Data to visualize

saveDir: str Path of directories to save the object to

dataName: str Label to include in the file name

minNumberOfNonzeroPoints: int, Default 5 Minimum number of non-zero points in signal to use it

oversamplingRate: int, Default 100 Periodogram oversampling rate

figsize: tuple, Default (8,8) Size of the figure in inches

title1: str, Default 'TimeSeries Data' Text of the upper title

title2: str, Default 'Lomb-Scargle periodogram' Text of the lower title

extension: str, Default '.png' Path of directories to save the object to

dpi: int, Default 300 Figure resolution if rasterized

Returns: None

Usage: makeLombScarglePeriodograms(df, '/dir1', 'myData')

addVisibilityGraph(*data, times, dataName='G1S1', coords=[0.05, 0.95, 0.05, 0.95], numberOfVGs=1, groups_ac_colors=['b'], fig=None, numberOfCommunities=6, printCommunities=False, fontsize=None, nodesize=None, level=0.55, commLineWidth=0.5, lineWidth=1.0, withLabel=True, withTitle=False, layout='circle', radius=0.07, noplot=False, horizontal=False*)

Draw a Visibility graph of data on a provided Matplotlib figure. We represent each timepoint in a series as a node. Temporal events are detected and indicated with solid blue lines encompassing groups of points, or communities. The shortest path identifies nodes (i.e. timepoints) that display high intensity, and thus dominate the global signal profile, are robust to noise, and are likely drivers of the global temporal behavior.

Parameters:

data: 2d numpy.array Array of data to visualize

times: 1d numpy.array Times corresponding to each data point, used for labels

dataName: str, Default 'G1S1' label to include in file name

coords: list, Default [0.05,0.95,0.05,0.95] Coordinates of location of the plot on the figure

numberOfVGs: int, Default 1 Number of plots to add to this figure

groups_ac_colors: list, Default ['b'] Colors corresponding to different groups of graphs

fig: matplotlib.figure, Default None Figure object

numberOfCommunities: int, Default 6 Number of communities

printCommunities: boolean, Default False Whether to print communities details to screen

fontsize: float, Default None Size of labels

nodesize: float, Default None Size of nodes

level: float, Default 0.55 Distance of the community lines to nodes

commLineWidth: float, Default 0.5 Width of the community lines

lineWidth: float, Default 1.0 Width of the edges between nodes

withLabel: boolean, Default True Whether to include label on plot

withTitle: boolean, Default False Whether to include title on plot

layout: str, Default 'circle' Type of the layout. Other option is 'line'

radius: float, Default 0.07 Radius of the circle

noplot: boolean, Default False Whether to make a plot or only calculate communities

Returns:

tuple (graph_nx, data, communities)

Usage:

addVisibilityGraph(exampleData, exampleTimes, fig=fig, fontsize=16, nodesize=700, level=0.85, commLineWidth=3.0, lineWidth=2.0, withLabel=False)

makeVisibilityGraph(*intensities, positions, saveDir, fileName, fontsize=16, nodesize=500, level=0.5, commLineWidth=3.0, lineWidth=2.0, layout='circle', horizontal=False, radius=0.03, figsize=(10, 10), addColorbar=True, colorbarAxisCoordinates=[0.9, 0.7, 0.02, 0.2], colorbarLabelsize=12, colorbarPrecision=2, extension='.png', dpi=300*)

Make either horizonral or normal visibility graph of a time series using function addVisibilityGraph. We represent each timepoint in a series as a node. Temporal events are detected and indicated with solid blue lines encompassing groups of points, or communities. The shortest path identifies nodes (i.e. timepoints) that display

high intensity, and thus dominate the global signal profile, are robust to noise, and are likely drivers of the global temporal behavior.

Parameters:

intensities: Data to plot

positions: Time points corresponding to data

saveDir: str Path of directories to save the object to

dataName: str Label to include in the file name

fontsize: float, Default 16 Labels fontsize

nodesize: int, Default 500 Node size

level: float, Default 0.5 Level

commLineWidth: float, Default 3.0 Communities lines width

lineWidth: float, Default 2.0 Edge lines width

layout: str, Default 'circle' Type of layout, 'circle' or 'line'

horizontal: boolean, Default False Whether to make horizontal of normal visibility graph

radius: float, Default 0.03 Rounding of the lines

figsize: tuple, Default (10,10) Figure size in inches

addColorbar: boolean, Default True Whether to add colorbar

colorbarAxisCoordinates: list, Default [0.90,0.7,0.02,0.2] colorbar axis coordinates

colorbarLabelsize: float, Default 12 Colorbar labels size

colorbarPrecision: int, Default 2 colorbar labels rounding

extension: str, Default '.png' Figure extension

dpi: int, Default 300 Figure resolution

Returns: None

Usage: makeVisibilityGraph(data, times, 'dir1/', 'myData')

makeVisibilityBarGraph(*data, times, saveDir, fileName, horizontal=False, barWidth=0.2, dotColor='b', barColor='r', arrowColor='k', id='', extension='.png', figsize=(8, 4), dpi=300*)

Bar-plot style visibility graph. Representing the intensities as bars, this is equivalent to connecting the top of each bar to another top if there is a direct line-of-sight to that top. The resulting visibility graph has characteristics that reflect the equivalent time series temporal structure and can be used to identify trends.

Parameters:

data: 2d numpy.array Numpy array of floats

times: 2d numpy.array Numpy array of floats

fileName: str Path where to save the figure file

fileName: str Name of the figure file to save

horizontal: boolean, default False Horizontal or normal visibility graph

barWidth: float, default 0.2 Horizontal or normal visibility graph

dotColor: str, default 'b' Color of the data points

barColor: str, default 'r' Color of the bars

arrowColor: str, default 'k' Color of lines

id: str or int, default '' Label to add to the figure title

extension: str, Default '.png' Figure format

figsize: tuple of int, Default (8,4) Figure size in inches

dpi: int, Default 300 Figure resolution

Returns: None

Usage: makeVisibilityBarGraph(A, data, times, 'my_figure')

makePlotOfPeak (*data_all*, *scores*, *selected_peak*, *selected_peak_value*, *plotID*)
Plot peaks. Function used internally during certain data processing steps

addColorbarToFigure (*fig, data, axisCoordinates=[0.9, 0.7, 0.02, 0.2], cmap=None, norm=None, labelsize=12, precision=2*)
Add colorbar to figure

Parameters:

fig: matplotlib.figure Data to plot

cmap: matplotlib.colors.LinearSegmentedColormap, Default None Colormap to use

norm: matplotlib.colors.Normalize, Default None Colormap normalization

axisCoordinates: list, Default [0.90,0.7,0.02,0.2] colorbar axis coordinates

labelsize: float, Default 12 Colorbar labels size

precision: int, Default 2 Colorbar labels rounding

Returns: None

Usage: addColorbarToFigure(fig, data)

makeDendrogramHeatmapOfClusteringObject (*ClusteringObject*, *saveDir*, *dataName*, *AutocorrNotPeriodogr=True*, *textScale=1.0*, *figsize=(12, 8)*, *extension='.png'*, *dpi=300*)
Make Dendrogram-Heatmap plot along with VIsibility graphs.

Parameters:

ClusteringObject: Clustering object

saveDir: str Path of directories to save the object to

dataName: str Label to include in the file name

AutocorrNotPeriodogr: boolean, Default True Whether to use autocorrelation method instead of periodograms

textScale: float, Default 1.0 scaling of text size

figsize: tuple, Default (12,8) Figure size in inches

extension: str, Default '.png' Figure format extension

dpi: int, Default 300 Figure resolution

Returns: None

Usage: makeDendrogramHeatmap(myObj, '/dir1', 'myData', AutocorrNotPeriodogr=True)

3.8 Utility functions

Submodule **pyiomica.utilityFunctions**

Utility functions

Functions

createDirectories(path)	Create a path of directories, unless the path already exists.
createReverseDictionary(inputDictionary)	Efficient way to create a reverse dictionary from a dictionary.
readMathIOmicaData(fileName)	Read text files exported by MathIOmica and convert to Python data
runCPUs(NumberOfAvailableCPUs, func, ...)	Parallelize function call with multiprocessing.Pool.

readMathIOmicaData (*fileName*)
Read text files exported by MathIOmica and convert to Python data

Parameters:

fileName: str Path of directories and name of the file containing data

Returns:

data Python data

Usage: data = readMathIOmicaData("../../MathIOmica/MathIOmica/MathIOmicaData/ExampleData/rnaExample")

runCPUs (*NumberOfAvailableCPUs*, *func*, *list_of_tuples_of_func_params*)
Parallelize function call with multiprocessing.Pool.

Parameters:

NumberOfAvailableCPUs: int Number of processes to create

func: function Function to apply, must take at most one argument

list_of_tuples_of_func_params: list Function parameters

Returns:

2d numpy.array Results of func in a numpy array

Usage: results = runCPUs(4, pAutocorrelation, [(times[i], data[i], allTimes) for i in range(10)])

createReverseDictionary (*inputDictionary*)
Efficient way to create a reverse dictionary from a dictionary. Utilizes Pandas.Dataframe.groupby and Numpy arrays indexing.

Parameters:

inputDictionary: dictionary Dictionary to reverse

Returns:

dictionary Reversed dictionary

Usage: revDict = createReverseDictionary(Dict)

createDirectories (*path*)
Create a path of directories, unless the path already exists.

Parameters:

path: str Path directory

Returns: None

Usage: createDirectories("/pathToFolder1/pathToSubFolder2")

CHAPTER 4

Dependencies

This graph was generated with **Python** module dependency visualization tool `pydeps`, see GitHub, by running the following (after installation of the necessary components):

```
pydeps --reverse --max-bacon 2 --pylib pyiomica
```

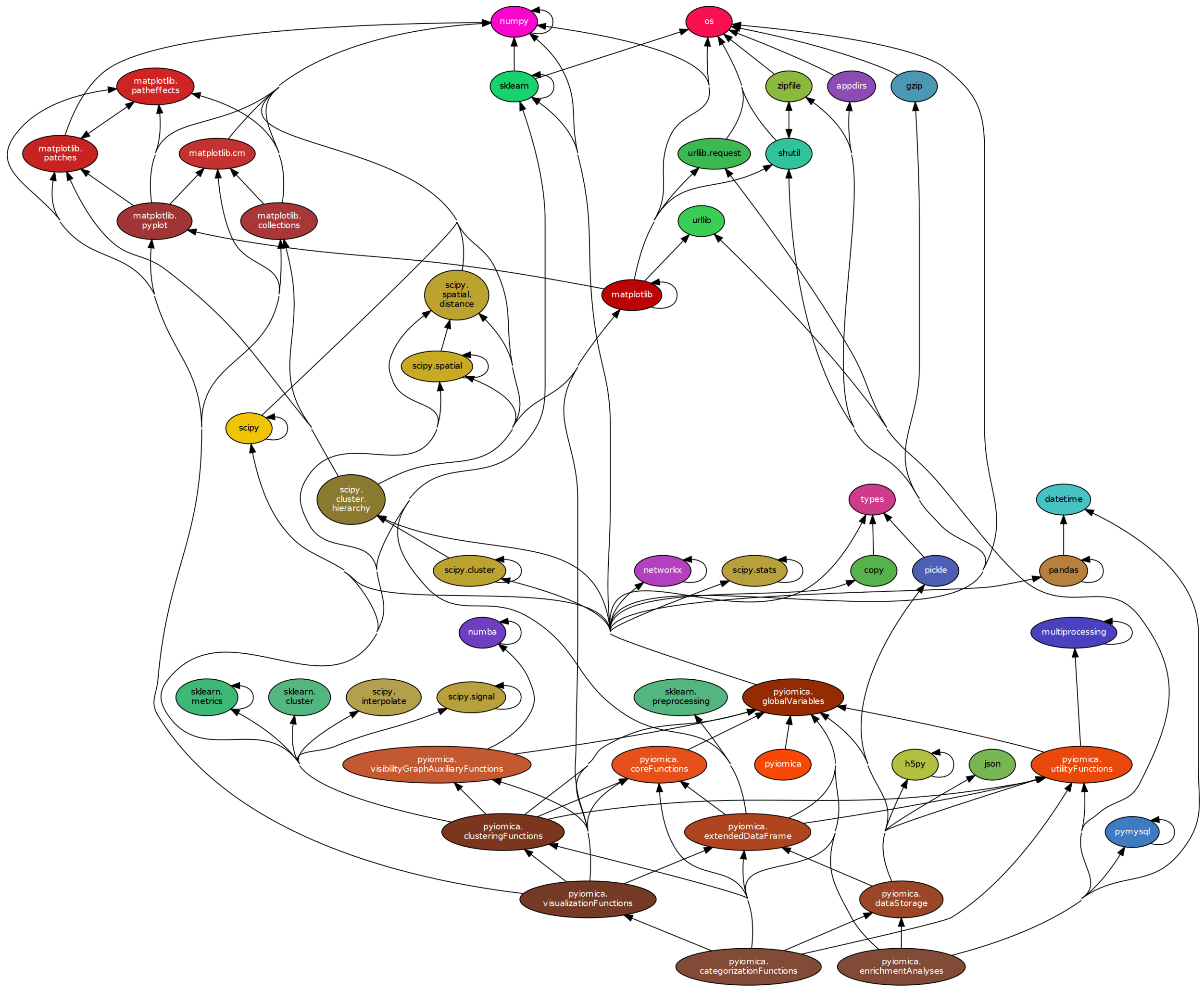

View raw graph

CHAPTER 5

Included data

PyIOmica is supplied with a set of examples that require folder data described here. This folder is created during installation of PyIOmica, see section Installation, and its location is stored in PyIOmica's global variable `ConstantPyIOmicaDataDirectory`.

Annotation and Enumeration functions such as Enrichment Analyses functions require various dictionaties and information files to be downloaded from the Internet. We provide a set of such dictionaries and files (in PyIOmica's data directory described above) to make it possible for user run PyIOmica in the abscence of the Internet connection. However the user can always override these files with updates ones by specifying proper flags to the functions that use the dictionaries.

CHAPTER 6

Examples

Examples of different uses of PyIOmica package API.

A Jupyter notebook with all examples is available at: https://github.com/gmiaslab/pyiomica/tree/master/docs/examples

6.1 Enrichment report

Function `ExportEnrichmentReport` generates enrichment report in form of ".xlsx" file.

	A	B	C	D	E	F	G	H	I	J
1		p-Value	BH-corrected p-Value	Significant	Counts in list	Counts in family	Total members	Counts in members	Description	List of gene hits
2	GO:0051092	9.89839E-11	5.35503E-08	TRUE	20	151	19774	7	positive regulation of NF-kappaB transcription factor activity, biological_process	TAB1 | MALT1 | TIRAP | CHUK | NFKB1 | NFKB2 | IRAK4
3	GO:0070498	3.37091E-08	9.11831E-06	TRUE	20	95	19774	5	interleukin-1-mediated signaling pathway, biological_process	TAB1 | CHUK | NFKB1 | NFKBIA | IRAK4
4	GO:0002223	7.72621E-08	1.19307E-05	TRUE	20	112	19774	5	stimulatory C-type lectin receptor signaling pathway, biological_process	TAB1 | MALT1 | CHUK | LYN | NFKB1
5	GO:0033209	8.8212E-08	1.19307E-05	TRUE	20	115	19774	5	tumor necrosis factor-mediated signaling pathway, biological_process	TNFSF13B | CHUK | TNFRSF13C | LTBR | NFKBIA
6	GO:0038095	6.36629E-07	5.81544E-05	TRUE	20	171	19774	5	Fc-epsilon receptor signaling pathway, biological_process	TAB1 | MALT1 | CHUK | LYN | NFKB1
7	GO:0043123	7.98199E-07	5.81544E-05	TRUE	20	179	19774	5	positive regulation of I-kappaB kinase/NF-kappaB signaling, biological_process	MALT1 | TIRAP | CHUK | LTBR | IRAK4
8	GO:0004674	1.18244E-06	5.81544E-05	TRUE	20	365	19774	6	protein serine/threonine kinase activity, molecular_function	CHUK | CSNK2A1 | CSNK2A2 | CSNK2B | ATM | IRAK4
9	GO:0006915	7.1017E-07	5.81544E-05	TRUE	20	551	19774	7	apoptotic process, biological_process	PARP1 | CSNK2A1 | CSNK2A2 | LTBR | GADD45B | NFKB1 | NFKBIA
10	GO:0001782	1.16322E-06	5.81544E-05	TRUE	20	21	19774	3	B cell homeostasis, biological_process	TNFSF13B | TNFRSF13C | LYN
11	GO:1905818	9.71958E-07	5.81544E-05	TRUE	20	2	19774	2	regulation of chromosome separation, biological_process	CSNK2A1 | CSNK2A2
12	GO:0033256	9.71958E-07	5.81544E-05	TRUE	20	2	19774	2	I-kappaB/NF-kappaB complex, cellular_component	NFKB1 | NFKBIA
13	GO:0005956	2.91395E-06	0.000105097	TRUE	20	3	19774	2	protein kinase CK2 complex, cellular_component	CSNK2A1 | CSNK2B
14	GO:0031296	2.91395E-06	0.000105097	TRUE	20	3	19774	2	B cell costimulation, biological_process	TNFSF13B | TNFRSF13C
15	GO:0002636	2.91395E-06	0.000105097	TRUE	20	3	19774	2	positive regulation of germinal center formation, biological_process	TNFSF13B | TNFRSF13C
16	GO:0031519	2.54827E-06	0.000105097	TRUE	20	27	19774	3	PcG protein complex, cellular_component	CSNK2A1 | CSNK2A2 | CSNK2B
17	GO:0047485	3.53757E-06	0.000119614	TRUE	20	106	19774	4	protein N-terminus binding, molecular_function	PARP1 | CSNK2A1 | CSNK2A2 | ATM
18	GO:0042802	4.498E-06	0.00013482	TRUE	20	1054	19774	8	identical protein binding, molecular_function	MALT1 | TIRAP | PARP1 | CSNK2A1 | CSNK2B | LTBR | NFKB1 | NFKBIA
19	GO:0002755	4.7349E-06	0.00013482	TRUE	20	33	19774	3	MyD88-dependent toll-like receptor signaling pathway, biological_process	TAB1 | TIRAP | IRAK4
20	GO:0006656	4.7349E-06	0.00013482	TRUE	20	33	19774	3	phosphatidylcholine biosynthetic process, biological_process	CSNK2A1 | CSNK2A2 | CSNK2B
21	GO:0031663	5.18976E-06	0.000140383	TRUE	20	34	19774	3	lipopolysaccharide-mediated signaling pathway, biological_process	MALT1 | LYN | NFKBIA
22	GO:1901796	8.20936E-06	0.000211489	TRUE	20	131	19774	4	regulation of signal transduction by p53 class mediator, biological_process	CSNK2A1 | CSNK2A2 | CSNK2B | ATM
23	GO:0030890	9.20351E-06	0.000226323	TRUE	20	41	19774	3	positive regulation of B cell proliferation, biological_process	TNFSF13B | TIRAP | TNFRSF13C
24	GO:0005515	1.23024E-05	0.000289374	TRUE	20	9629	19774	19	protein binding, molecular_function	TAB1 | TNFSF13B | MALT1 | TIRAP | CHUK | PARP1 | CSNK2A1 | CSNK2A2 | CSNK2B |
25	GO:0018105	1.80798E-05	0.000407548	TRUE	20	160	19774	4	peptidyl-serine phosphorylation, biological_process	CHUK | CSNK2A1 | CSNK2A2 | ATM

List

The are following columns in each Worksheet of the output ".xlsx" file

- ID: Annotation term identifier
- p-Value: Probability to find at least "Counts in members" number of genes when drawing "Counts in list" genes "Counts in family" times (without replacement) from "Counts in members" identifiers. Note: this is the behavior when using default setting, i.e. Hypergeometric distribution testing function

- BH-corrected p-Value: p-Value corrected for false discovery rate (FDR) via Benjamini-Hochberg procedure
- Significant: Whether the "BH-corrected p-Value" is below the threshold (typically 0.05) specified for the enrichment analysis
- Counts in list: Number of genes in the input list
- Counts in family: Number of genes in a particular annotation term
- Total members: Number of members (e.g. UniprotIDs) in the IDs to annotation terms dictionary
- Counts in members: Number of annotation terms in which at least of gene from the input list appear
- Description: Description of a particular annotation term, e.g. details/type
- List of gene hits: Gene identifiers that are found in a particular annotation term. The identifiers are separated by a Vertical bar

6.2 Import of MathIOmica Objects

```
#import sys
#sys.path.append("../..")

import pyiomica as pio

from pyiomica.utilityFunctions import readMathIOmicaData

print(pio.ConstantPyIOmicaExamplesDirectory, '\n')
tempPath = pio.os.path.join(pio.ConstantPyIOmicaExamplesDirectory, 'MathIOmicaExamples
↪')

rnaExample = readMathIOmicaData(pio.os.path.join(tempPath, 'rnaExample'))
print('rnaExample:', str(rnaExample)[:400], '\t. . .\n')

proteinClassificationExample = readMathIOmicaData(pio.os.path.join(tempPath,
↪'proteinClassificationExample'))
print('proteinClassificationExample:', str(proteinClassificationExample)[:400], '\t. .
↪ .\n')

proteinTimeSeriesExample = readMathIOmicaData(pio.os.path.join(tempPath,
↪'proteinTimeSeriesExample'))
print('proteinTimeSeriesExample:', str(proteinTimeSeriesExample)[:400], '\t. . .\n')

proteinExample = readMathIOmicaData(pio.os.path.join(tempPath, 'proteinExample'))
print('proteinExample:', str(proteinExample)[:400], '\t. . .\n')

metabolomicsPositiveModeExample = readMathIOmicaData(pio.os.path.join(tempPath,
↪'metabolomicsPositiveModeExample'))
print('metabolomicsPositiveModeExample:', str(metabolomicsPositiveModeExample)[:400],
↪'\t. . .\n')

metabolomicsNegativeModeExample = readMathIOmicaData(pio.os.path.join(tempPath,
↪'metabolomicsNegativeModeExample'))
print('metabolomicsNegativeModeExample:', str(metabolomicsNegativeModeExample)[:400],
↪'\t. . .\n')
```

6.3 Clustering object export example

Example of a clustering object exported to ".xlsx" file.

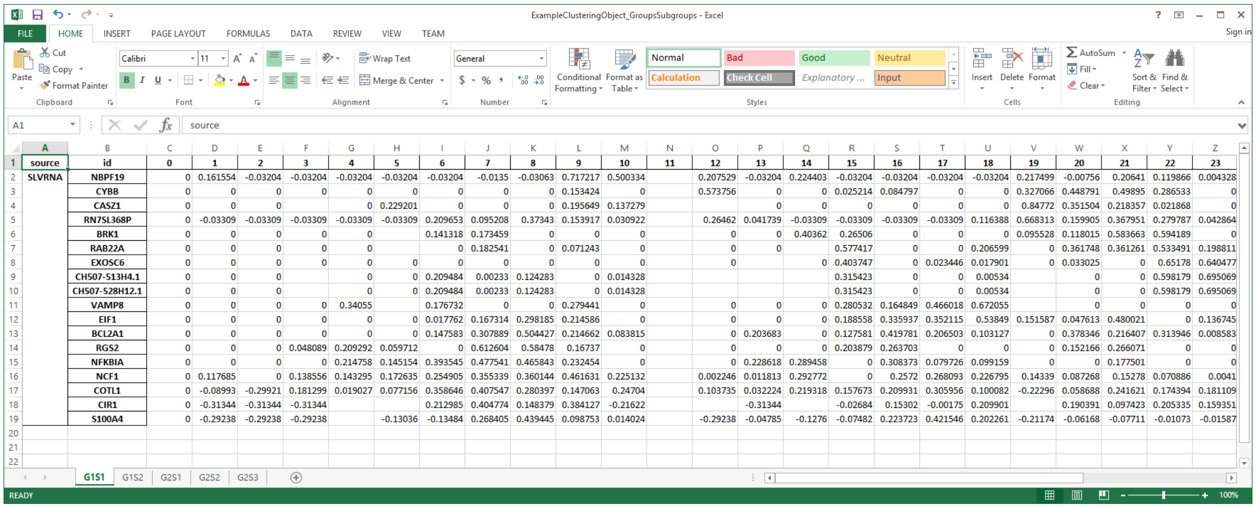

source	id	0	1	2	3	4	5	6	7	8	9	10	11	12	13	14	15	16	17	18	19	20	21	22	23
SLVRNA	NBPF19	0	0.161554	-0.03204	-0.03204	-0.03204	-0.03204	-0.03204	-0.0135	-0.03063	0.717217	0.500334		0.207529	-0.03204	0.224403	-0.03204	-0.03204	-0.03204	-0.03204	0.217499	-0.00756	0.20641	0.119866	0.004328
	CYBB	0	0	0	0	0	0	0	0	0	0.153424	0		0.573756	0	0	0.025214	0.084797	0	0	0.327066	0.448791	0.49895	0.286533	0
	CASZ1	0	0	0		0	0.229201	0	0	0	0.195649	0.137279			0	0	0	0	0	0	0.84772	0.351504	0.218357	0.021868	0
	RN7SL368P	0	-0.03309	-0.03309	-0.03309	-0.03309	-0.03309	0.209653	0.095208	0.37343	0.153917	0.030922		0.26462	0.041739	-0.03309	-0.03309	-0.03309	-0.03309	0.116388	0.668313	0.159905	0.367951	0.279787	0.042864
	BRK1	0	0	0	0	0		0.141318	0.173459	0	0	0		0	0	0.40362	0.26506	0	0	0	0.095528	0.118015	0.583663	0.594189	0
	RAB22A	0	0	0	0	0		0	0.182541	0	0.071243	0		0	0		0.577417	0	0	0.206599	0	0.361748	0.361261	0.533491	0.198811
	EXOSC6	0	0	0	0	0	0	0	0	0	0	0		0		0	0.403747	0	0.023446	0.017901	0	0.033025	0	0.65178	0.640477
	CH507-513H4.1	0	0	0		0	0	0.209484	0.00233	0.124283	0	0.014328					0.315423	0	0	0.00534		0	0	0.598179	0.695069
	CH507-528H12.1	0	0	0		0	0	0.209484	0.00233	0.124283	0	0.014328					0.315423	0	0	0.00534		0	0	0.598179	0.695069
	VAMP8	0	0	0	0	0.34055		0.176732	0	0	0.279441	0		0	0	0	0.280532	0.164849	0.466018	0.672055		0	0	0	0
	EIF1	0	0	0	0	0	0	0.017762	0.167314	0.298185	0.214586	0		0	0	0	0.188558	0.335937	0.352115	0.53849	0.151587	0.047613	0.480021	0	0.136745
	BCL2A1	0	0	0	0	0	0	0.147583	0.307889	0.504427	0.214662	0.083815		0	0.203683	0	0.127581	0.419781	0.206503	0.103127	0	0.378346	0.216407	0.313946	0.008583
	RGS2	0	0	0	0.048089	0.209292	0.059712	0	0.612604	0.58478	0.16737	0		0	0	0	0.203879	0.263703	0	0	0	0.152166	0.266071	0	0
	NFKBIA	0	0	0	0	0.214758	0.145154	0.393545	0.477541	0.465843	0.232454	0		0	0.228618	0.289458	0	0.308373	0.079726	0.099159	0	0	0.177501	0	0
	NCF1	0	0.117685	0	0.138556	0.143295	0.172635	0.254905	0.355339	0.360144	0.461631	0.225132		0.002246	0.011813	0.292772	0	0.2572	0.268093	0.226795	0.14339	0.087268	0.15278	0.070886	0.0041
	COTL1	0	-0.08993	-0.29921	0.181299	0.019027	0.077156	0.358646	0.407547	0.280397	0.147063	0.24704		0.103735	0.032224	0.219318	0.157673	0.209931	0.305956	0.100082	-0.22296	0.058688	0.241621	0.174394	0.181109
	CIR1	0	-0.31344	-0.31344	-0.31344			0.212985	0.404774	0.148379	0.384127	-0.21622			-0.31344		-0.02684	0.15302	-0.00175	0.209901		0.190391	0.097423	0.205335	0.159351
	S100A4	0	-0.29238	-0.29238	-0.29238		-0.13036	-0.13484	0.268405	0.439445	0.098753	0.014024		-0.29238	-0.04785	-0.1276	-0.07482	0.223723	0.421546	0.202261	-0.21174	-0.06168	-0.07711	-0.01073	-0.01587

The file contains one Spreadsheet for each subgroup in a clustering object. The spreadsheet is structrured as follows. The first column indicates the data source, the second column has gene identifiers, all the remaining colummns contain data and autocorrelations. Note, this is the structure when using the default settings. User can modify output by setting various options.

6.4 GO Analysis examples

```
#import sys
#sys.path.append("../..")

import pyiomica as pio

from pyiomica.enrichmentAnalyses import GOAnalysis, ExportEnrichmentReport
from pyiomica import dataStorage as ds

EnrichmentOutputDirectory = 'results/EnrichmentOutputDirectory/'

#Let's do a GO analysis for a group of genes, annotated with their "Gene Symbol":
goExample1 = GOAnalysis(["TAB1", "TNFSF13B", "MALT1", "TIRAP", "CHUK",
                                "TNFRSF13C", "PARP1", "CSNK2A1", "CSNK2A2", "CSNK2B",
↪"LTBR",
                                "LYN", "MYD88", "GADD45B", "ATM", "NFKB1", "NFKB2",
↪"NFKBIA",
                                "IRAK4", "PIAS4", "PLAU"])

ExportEnrichmentReport(goExample1, AppendString='goExample1',␣
↪OutputDirectory=EnrichmentOutputDirectory + 'GOAnalysis/')

#The information can be computed for multiple groups, if these are provided as an␣
↪association:
analysisGOAssociation = GOAnalysis({"Group1": ["C6orf57", "CD46", "DHX58", "HMGB3",
↪"MAP3K5", "NFKB2", "NOS2", "PYCARD", "PYDC1", "SSC5D"],
                                     "Group2": ["TAB1", "TNFSF13B", "MALT1
↪", "TIRAP", "CHUK", "TNFRSF13C", "PARP1", "CSNK2A1", "CSNK2A2", "CSNK2B", "LTBR",
```

(continues on next page)

(continued from previous page)

```
                                                                    "LYN", "MYD88", "GADD45B",
↪ "ATM", "NFKB1", "NFKB2", "NFKBIA", "IRAK4", "PIAS4", "PLAU"]})

ExportEnrichmentReport(analysisGOAssociation, AppendString='analysisGOAssociation',␣
↪OutputDirectory=EnrichmentOutputDirectory + 'GOAnalysis/')

#The data can be computed with or without a label. If labeled, the gene ID must be␣
↪the first element for each ID provided. The data is in the form {ID,label}:
analysisGOLabel = GOAnalysis([["C6orf57", "Protein"], ["CD46", "Protein"], ["DHX58",
↪"Protein"], ["HMGB3", "Protein"], ["MAP3K5", "Protein"],
                                        ["NFKB2", "Protein"], ["NOS2", "Protein"], [
↪"PYCARD", "Protein"], ["PYDC1","Protein"], ["SSC5D", "Protein"]])

ExportEnrichmentReport(analysisGOLabel, AppendString='analysisGOLabel',␣
↪OutputDirectory=EnrichmentOutputDirectory + 'GOAnalysis/')

#The data can be mixed, e.g. proteins and RNA with different labels:
analysisGOMixed = GOAnalysis([["C6orf57", "Protein"], ["CD46", "Protein"], ["DHX58",
↪"Protein"], ["HMGB3", "RNA"], ["HMGB3", "Protein"], ["MAP3K5", "Protein"],
                                        ["NFKB2", "RNA"], ["NFKB2", "Protein"], ["NOS2
↪", "RNA"], ["PYCARD", "RNA"], ["PYDC1", "Protein"], ["SSC5D", "Protein"]])

ExportEnrichmentReport(analysisGOMixed, AppendString='analysisGOMixed',␣
↪OutputDirectory=EnrichmentOutputDirectory + 'GOAnalysis/')

#We can instead treat the data as different by setting the MultipleList and␣
↪MultipleListCorrection options:
analysisGOMixedMulti = GOAnalysis([["C6orf57", "Protein"], ["CD46", "Protein"], [
↪"DHX58", "Protein"], ["HMGB3", "RNA"], ["HMGB3", "Protein"], ["MAP3K5", "Protein"],
                                            ["NFKB2", "RNA"], ["NFKB2", "Protein"], [
↪"NOS2", "RNA"], ["PYCARD", "RNA"], ["PYDC1", "Protein"], ["SSC5D", "Protein"]],
                                            MultipleList=True, MultipleListCorrection=
↪'Automatic')

ExportEnrichmentReport(analysisGOMixedMulti, AppendString='analysisGOMixedMulti',␣
↪OutputDirectory=EnrichmentOutputDirectory + 'GOAnalysis/')

#Let's consider an example from real protein data. We will use already clustered data,
↪ from the examples. Let's import the data:
ExampleClusteringObject = ds.read(pio.os.path.join(pio.
↪ConstantPyIOmicaExamplesDirectory, 'exampleClusteringObject_SLV_Delta_LAG1_Autocorr
↪'))

if not ExampleClusteringObject is None:
    #We calculate the GOAnalysis for each group in each class:
    ExampleClusteringObjectGO = GOAnalysis(ExampleClusteringObject,␣
↪MultipleListCorrection='Automatic')

    ExportEnrichmentReport(ExampleClusteringObjectGO, AppendString=
↪'ExampleClusteringObjectGO', OutputDirectory=EnrichmentOutputDirectory +
↪'GOAnalysis/')
```

6.5 KEGG Analysis examples

```
#import sys
#sys.path.append("../..")

import pyiomica as pio

from pyiomica.enrichmentAnalyses import KEGGAnalysis, ExportEnrichmentReport
from pyiomica import dataStorage as ds

EnrichmentOutputDirectory = pio.os.path.join('results', 'EnrichmentOutputDirectory', '
↪')

#Let's do a KEGG pathway analysis for a group of genes (most in the NFKB pathway),␣
↪annotated with their "Gene Symbol":
keggExample1 = KEGGAnalysis(["TAB1", "TNFSF13B", "MALT1", "TIRAP", "CHUK", "TNFRSF13C
↪", "PARP1", "CSNK2A1", "CSNK2A2", "CSNK2B", "LTBR", "LYN", "MYD88",
                                        "GADD45B", "ATM", "NFKB1", "NFKB2", "NFKBIA",
↪"IRAK4", "PIAS4", "PLAU", "POLR3B", "NME1", "CTPS1", "POLR3A"])

ExportEnrichmentReport(keggExample1, AppendString='keggExample1',␣
↪OutputDirectory=EnrichmentOutputDirectory + 'KEGGAnalysis/')

#The information can be computed for multiple groups, if these are provided as an␣
↪association:
analysisKEGGAssociation = KEGGAnalysis({"Group1": ["C6orf57", "CD46", "DHX58", "HMGB3
↪", "MAP3K5", "NFKB2", "NOS2", "PYCARD", "PYDC1", "SSC5D"],
                                                    "Group2": ["TAB1", "TNFSF13B",
↪"MALT1", "TIRAP", "CHUK", "TNFRSF13C", "PARP1", "CSNK2A1", "CSNK2A2", "CSNK2B",
↪"LTBR",
                                                "LYN", "MYD88", "GADD45B", "ATM",
↪"NFKB1", "NFKB2", "NFKBIA", "IRAK4", "PIAS4", "PLAU", "POLR3B", "NME1", "CTPS1",
↪"POLR3A"]})

ExportEnrichmentReport(analysisKEGGAssociation, AppendString='analysisKEGGAssociation
↪', OutputDirectory=EnrichmentOutputDirectory + 'KEGGAnalysis/')

#The data can be computed with or without a label. If labeled, the gene ID must be␣
↪the first element for each ID provided. The data is in the form {ID,label}:
analysisKEGGLabel = KEGGAnalysis([["C6orf57", "Protein"], ["CD46", "Protein"], ["DHX58
↪", "Protein"], ["HMGB3", "Protein"], ["MAP3K5", "Protein"],
                                            ["NFKB2", "Protein"], ["NOS2", "Protein"],
↪ ["PYCARD", "Protein"], ["PYDC1","Protein"], ["SSC5D", "Protein"]])

ExportEnrichmentReport(analysisKEGGLabel, AppendString='analysisKEGGLabel',␣
↪OutputDirectory=EnrichmentOutputDirectory + 'KEGGAnalysis/')

#The same result is obtained if IDs are enclosed in list brackets:
analysisKEGGNoLabel = KEGGAnalysis([["C6orf57"], ["CD46"], ["DHX58"], ["HMGB3"], [
↪"MAP3K5"], ["NFKB2"], ["NOS2"], ["PYCARD"], ["PYDC1"], ["SSC5D"]])

ExportEnrichmentReport(analysisKEGGNoLabel, AppendString='analysisKEGGNoLabel',␣
↪OutputDirectory=EnrichmentOutputDirectory + 'KEGGAnalysis/')

#The same result is obtained if IDs are input as strings:
analysisKEGGstrings = KEGGAnalysis(["C6orf57", "CD46", "DHX58", "HMGB3", "MAP3K5",
↪"NFKB2", "NOS2", "PYCARD", "PYDC1", "SSC5D"])
```

(continues on next page)

(continued from previous page)

```
ExportEnrichmentReport(analysisKEGGstrings, AppendString='analysisKEGGstrings',␣
↪OutputDirectory=EnrichmentOutputDirectory + 'KEGGAnalysis/')

#The data can be mixed, e.g. proteins and RNA with different labels:
analysisKEGGMixed = KEGGAnalysis([["C6orf57", "Protein"], ["CD46", "Protein"], ["DHX58
↪", "Protein"], ["HMGB3", "RNA"], ["HMGB3", "Protein"], ["MAP3K5", "Protein"],
                                            ["NFKB2", "RNA"], ["NFKB2", "Protein"], [
↪"NOS2", "RNA"], ["PYCARD", "RNA"], ["PYDC1", "Protein"], ["SSC5D", "Protein"]])

ExportEnrichmentReport(analysisKEGGMixed, AppendString='analysisKEGGMixed',␣
↪OutputDirectory=EnrichmentOutputDirectory + 'KEGGAnalysis/')

#The data in this case treated as originating from a single population. Protein and␣
↪RNA labeled data for the same identifier are treated as equivalent.
#We can instead treat the data as different by setting the MultipleList and␣
↪MultipleListCorrection options:
analysisKEGGMixedMulti = KEGGAnalysis([["C6orf57", "Protein"], ["CD46", "Protein"], [
↪"DHX58", "Protein"], ["HMGB3", "RNA"], ["HMGB3", "Protein"], ["MAP3K5", "Protein"],
                                                ["NFKB2", "RNA"], ["NFKB2", "Protein
↪"], ["NOS2", "RNA"], ["PYCARD", "RNA"], ["PYDC1", "Protein"], ["SSC5D", "Protein"]],
                                                MultipleList=True,␣
↪MultipleListCorrection='Automatic')

ExportEnrichmentReport(analysisKEGGMixedMulti, AppendString='analysisKEGGMixedMulti',␣
↪OutputDirectory=EnrichmentOutputDirectory + 'KEGGAnalysis/')

#We can carry out a "Molecular" analysis for compound data. We consider the following␣
↪metabolomics data, which has labels "Meta"
#and additional mass and retention time information in the form {identifier,mass,␣
↪retention time, label}:
compoundsExample = [["cpd:C19691", 325.2075, 10.677681, "Meta"], ["cpd:C17905", 594.
↪2002, 8.727458, "Meta"],["cpd:C09921", 204.0784, 12.3909445, "Meta"],
                    ["cpd:C18218", 272.2356, 13.473582, "Meta"], ["cpd:C14169", 235.
↪1573, 12.267084, "Meta"],["cpd:C14245", 262.2296, 13.545572, "Meta"],
                    ["cpd:C09137", 352.2615, 14.0554285, "Meta"], ["cpd:C09674", 296.
↪1624, 12.147417, "Meta"], ["cpd:C00449", 276.1334, 11.004139, "Meta"],
                    ["cpd:C02999", 364.1497, 12.147243, "Meta"], ["cpd:C07915", 309.
↪194, 7.3625283, "Meta"],["cpd:C08760", 496.2309, 8.7241125, "Meta"],
                    ["cpd:C14549", 276.0972, 11.078914, "Meta"], ["cpd:C20533", 601.
↪3378, 12.75722, "Meta"], ["cpd:C20790", 212.1051, 7.127666, "Meta"],
                    ["cpd:C09137", 352.2613, 12.869867, "Meta"], ["cpd:C17648", 400.
↪2085, 10.843841, "Meta"], ["cpd:C07807", 240.1471, 0.48564285, "Meta"],
                    ["cpd:C08564", 324.0948, 10.281, "Meta"], ["cpd:C19426", 338.2818,
↪ 13.758765, "Meta"], ["cpd:C02943", 468.3218, 14.263261, "Meta"],
                    ["cpd:C04882", 1193.342, 14.707576, "Meta"]]

compoundsExampleKEGG = KEGGAnalysis(compoundsExample, FilterSignificant=True,␣
↪AnalysisType='Molecular')

ExportEnrichmentReport(compoundsExampleKEGG, AppendString='compoundsExampleKEGG',␣
↪OutputDirectory=EnrichmentOutputDirectory + 'KEGGAnalysis/')

#We can carry out multiomics data analysis. We consider the following simple example:
multiOmicsData = [["C6orf57", "Protein"], ["CD46", "Protein"], ["DHX58", "Protein"], [
↪"HMGB3", "RNA"], ["HMGB3", "Protein"],
                    ["MAP3K5", "Protein"], ["NFKB2", "RNA"], ["NFKB2", "Protein"], [
↪"NOS2", "RNA"], ["PYCARD", "RNA"], ["PYDC1", "Protein"],
```

(continues on next page)

(continued from previous page)

```
                    ["SSC5D", "Protein"], ["cpd:C19691", 325.2075, 10.677681, "Meta"],
↪ ["cpd:C17905", 594.2002, 8.727458, "Meta"],
                    ["cpd:C09921", 204.0784, 12.3909445, "Meta"], ["cpd:C18218", 272.
↪2356, 13.473582, "Meta"],
                    ["cpd:C14169", 235.1573, 12.267084, "Meta"], ["cpd:C14245", 262.
↪2296, 13.545572, "Meta"],
                    ["cpd:C09137", 352.2615, 14.0554285, "Meta"], ["cpd:C09674", 296.
↪1624, 12.147417, "Meta"],
                    ["cpd:C00449", 276.1334, 11.004139, "Meta"],["cpd:C02999", 364.
↪1497, 12.147243, "Meta"],
                    ["cpd:C07915", 309.194, 7.3625283, "Meta"],["cpd:C08760", 496.
↪2309, 8.7241125, "Meta"],
                    ["cpd:C14549", 276.0972, 11.078914, "Meta"],["cpd:C20533", 601.
↪3378, 12.75722, "Meta"],
                    ["cpd:C20790", 212.1051, 7.127666, "Meta"], ["cpd:C09137", 352.
↪2613, 12.869867, "Meta"],
                    ["cpd:C17648", 400.2085, 10.843841, "Meta"], ["cpd:C07807", 240.
↪1471, 0.48564285, "Meta"],
                    ["cpd:C08564", 324.0948, 10.281, "Meta"], ["cpd:C19426", 338.2818,
↪ 13.758765, "Meta"],
                    ["cpd:C02943", 468.3218, 14.263261, "Meta"], ["cpd:C04882", 1193.
↪342, 14.707576, "Meta"]]

#We can carry out "Genomic" and "Molecular" analysis concurrently by setting␣
↪AnalysisType = "All":
multiOmicsDataKEGG = KEGGAnalysis(multiOmicsData, AnalysisType='All',␣
↪MultipleList=True, MultipleListCorrection='Automatic')

ExportEnrichmentReport(multiOmicsDataKEGG, AppendString='multiOmicsDataKEGG',␣
↪OutputDirectory=EnrichmentOutputDirectory + 'KEGGAnalysis/')

#Let's consider an example from real protein data. We will use already clustered data,
↪ from the examples. Let's import the data:
ExampleClusteringObject = ds.read(pio.os.path.join(pio.
↪ConstantPyIOmicaExamplesDirectory, 'exampleClusteringObject_SLV_Delta_LAG1_Autocorr
↪'))

if not ExampleClusteringObject is None:
    #We calculate the KEGGAnalysis for each group in each class:
    ExampleClusteringObject = KEGGAnalysis(ExampleClusteringObject)

    ExportEnrichmentReport(ExampleClusteringObject, AppendString=
↪'ExampleClusteringObject', OutputDirectory=EnrichmentOutputDirectory +
↪'KEGGAnalysis/')
```

6.6 Visibility Graph examples

We represent each timepoint in a series as a node. Temporal events are detected and indicated with solid blue lines encompassing groups of points, or communities. The shortest path identifies nodes (i.e. timepoints) that display high intensity, and thus dominate the global signal profile, are robust to noise, and are likely drivers of the global temporal behavior.

Representing the intensities as bars, this is equivalent to connecting the top of each bar to another top if there is a direct line-of-sight to that top. The resulting visibility graph has characteristics that reflect the equivalent time series

temporal structure and can be used to identify trends.

```
#import sys
#sys.path.append("../..")

import pyiomica as pio

from pyiomica.visualizationFunctions import makeVisibilityGraph,␣
↪makeVisibilityBarGraph
from pyiomica import dataStorage as ds
from pyiomica.extendedDataFrame import DataFrame

# Get an example of a clustering object from ConstantPyIOmicaExamplesDirectory
ExampleClusteringObject = ds.read(pio.os.path.join(pio.
↪ConstantPyIOmicaExamplesDirectory, 'exampleClusteringObject_SLV_Delta_LAG1_Autocorr
↪'))

# Use Group 1, Subgroup 2 data (chosen arbitrarily here)
df_data = ExampleClusteringObject[1][2]['data']

# Read intensities of all signals and the measurement time points (here positions)
intensities, positions = df_data.values, df_data.columns.values

# Normalize and aggregate the intensities to obtain one averaged signal
normalized_intensities = DataFrame(data=intensities).imputeMissingWithMedian().
↪apply(lambda data: pio.np.sum(data[data > 0.0]) / len(data), axis=0).values

# Make normal visibility graphs on a 'cirle' and 'line' layouts
makeVisibilityGraph(normalized_intensities, positions, 'results', 'circle_VG', layout=
↪'circle')
makeVisibilityGraph(normalized_intensities, positions, 'results', 'line_VG', layout=
↪'line')

# Make horizontal visibility graphs on a 'cirle' and 'line' layouts
makeVisibilityGraph(normalized_intensities, positions, 'results', 'circle_VG', layout=
↪'circle', horizontal=True)
makeVisibilityGraph(normalized_intensities, positions, 'results', 'line_VG', layout=
↪'line', horizontal=True)

# Make horizontal and normal bar-style visibility graphs
makeVisibilityBarGraph(normalized_intensities, positions, 'results', 'barVG')
makeVisibilityBarGraph(normalized_intensities, positions, 'results', 'barVG',␣
↪horizontal=True)
```

Normal (left) and Horizontal (right) visibility graph on a circular layout:

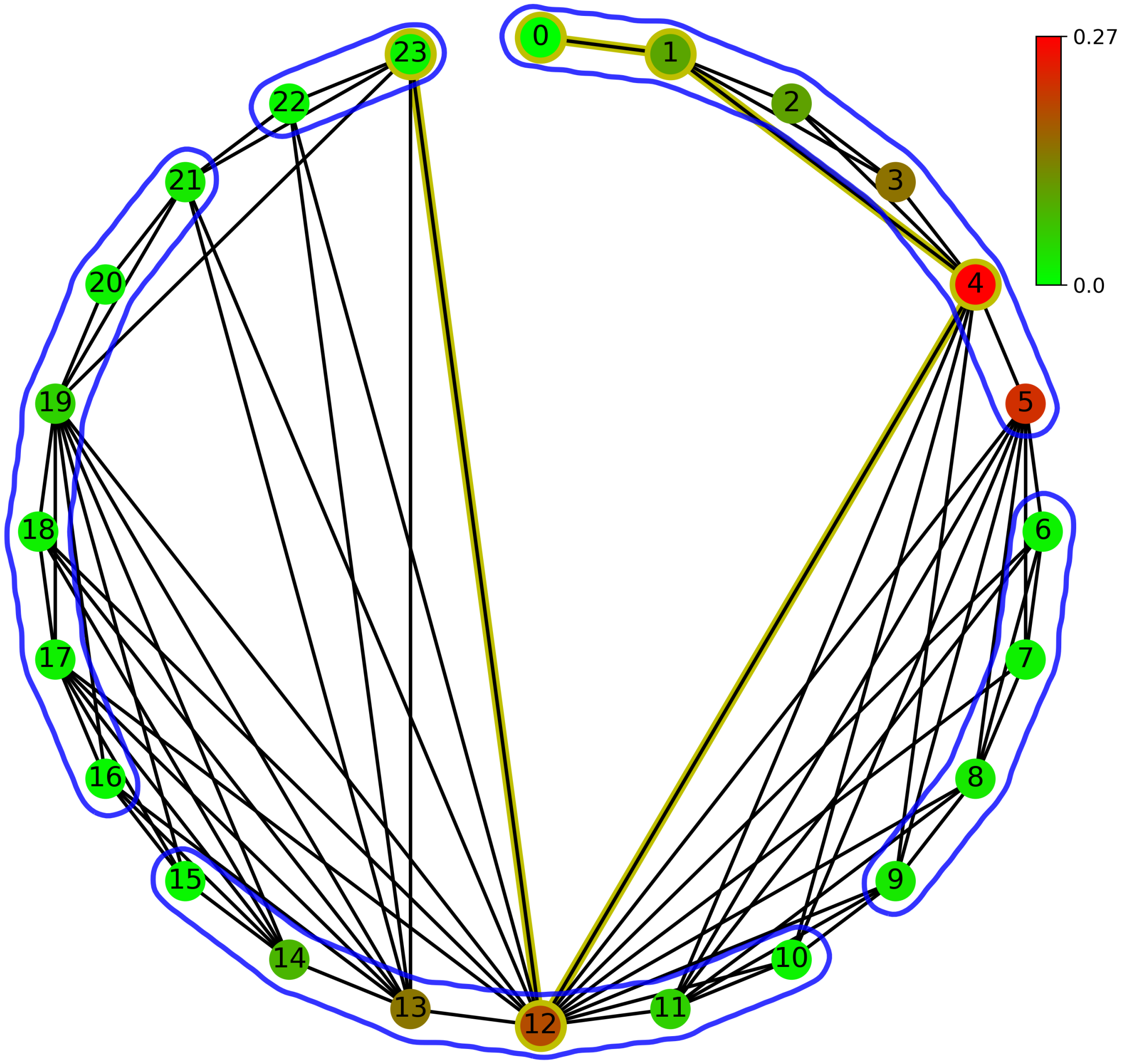
0
1
2
3
4
5
6
7
8
9
10
11
12
13
14
15
16
17
18
19
20
21
22
23
0.27
0.0

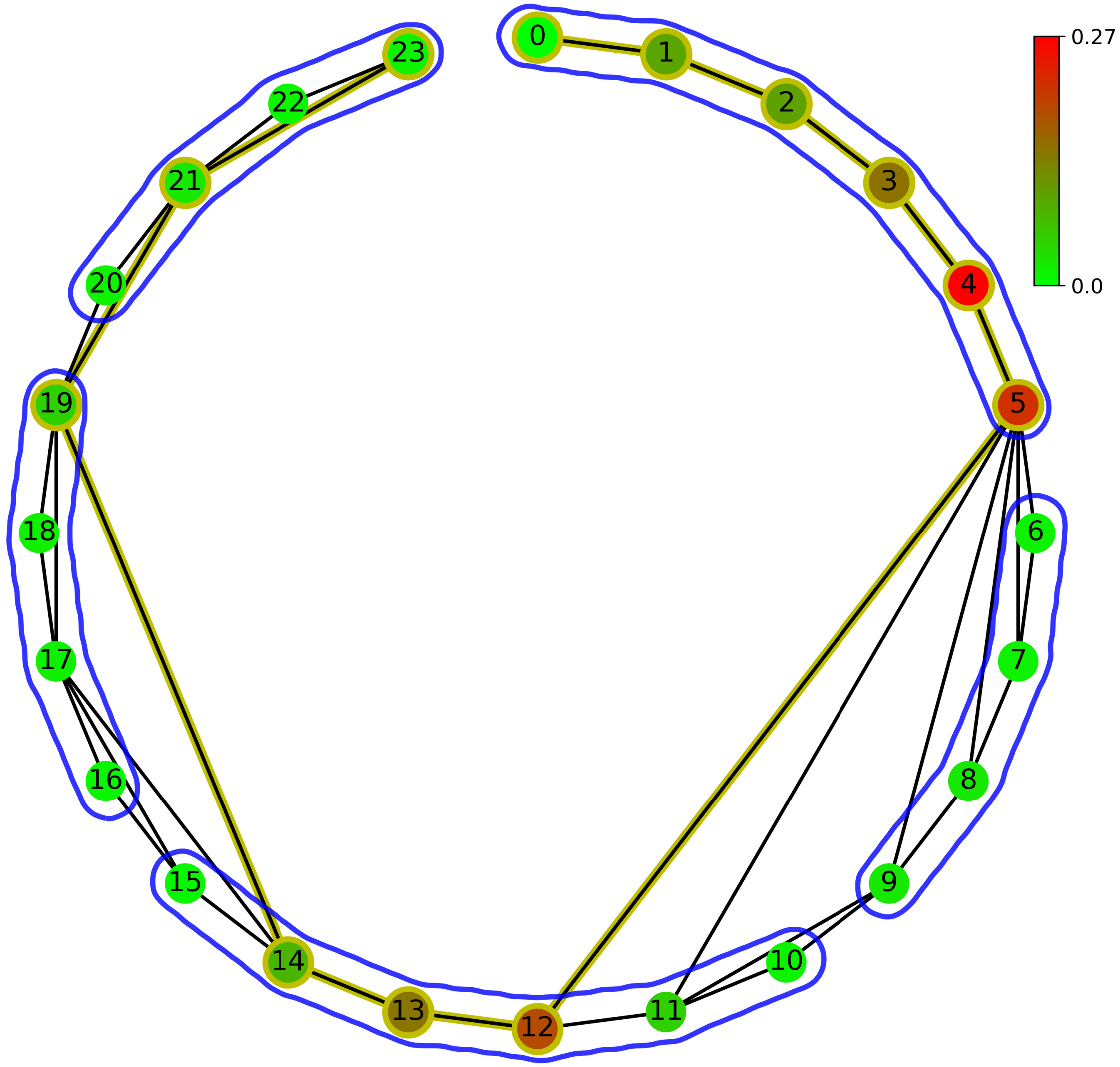

Normal (left) and Horizontal (right) visibility graph on a linear layout:

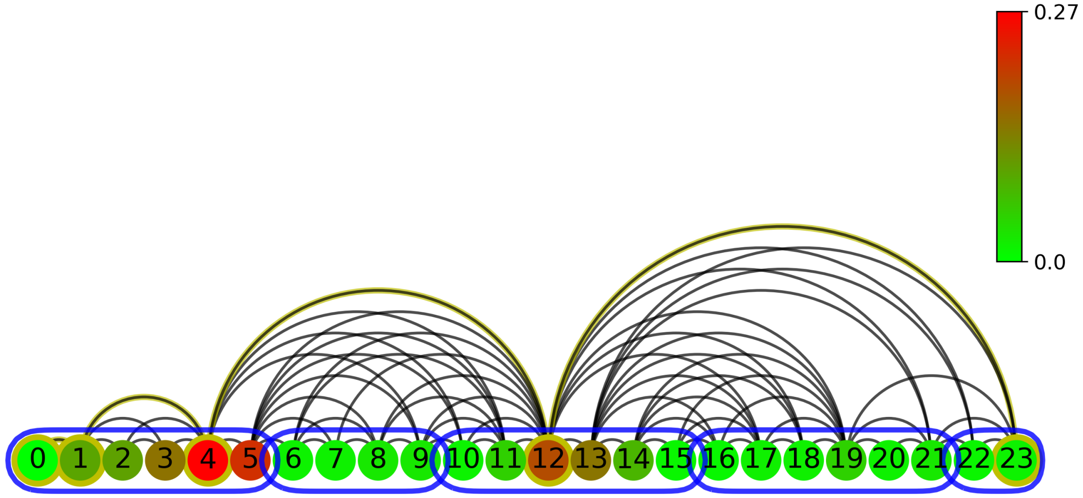
0.27
0.0
0
1
2
3
4
5
6
7
8
9
10
11
12
13
14
15
16
17
18
19
20
21
22
23

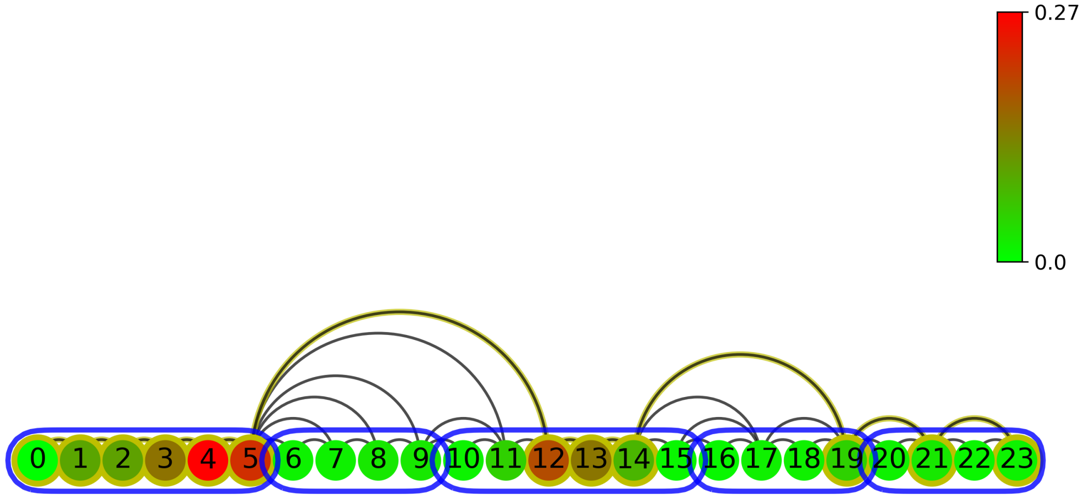

Normal (left) and Horizontal (right) bar-style visibility graph:

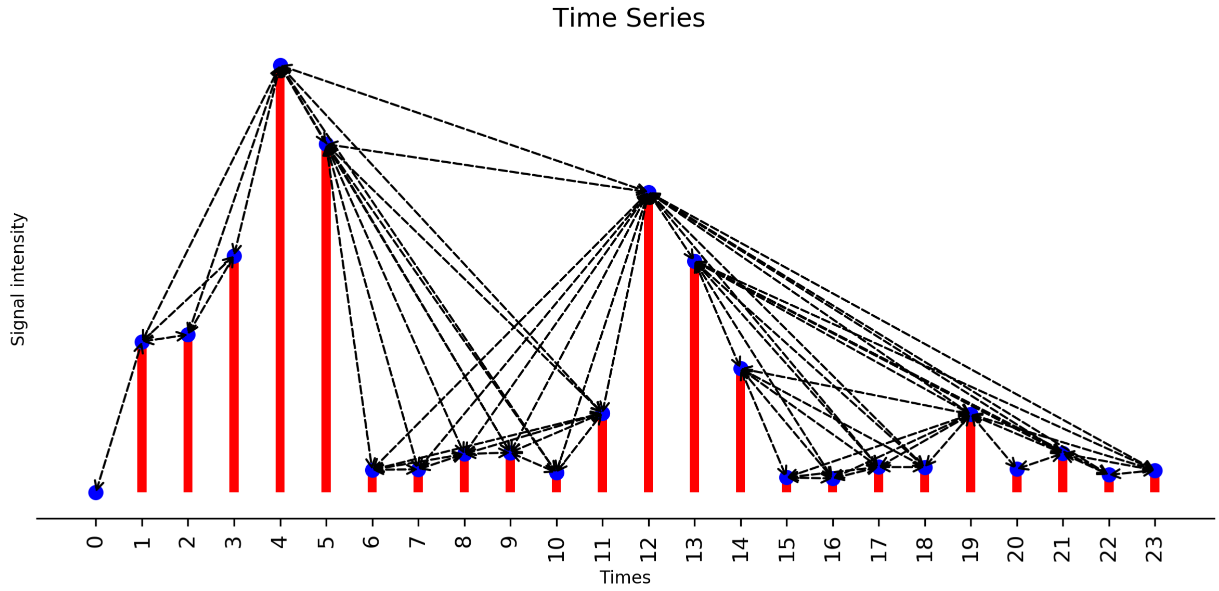

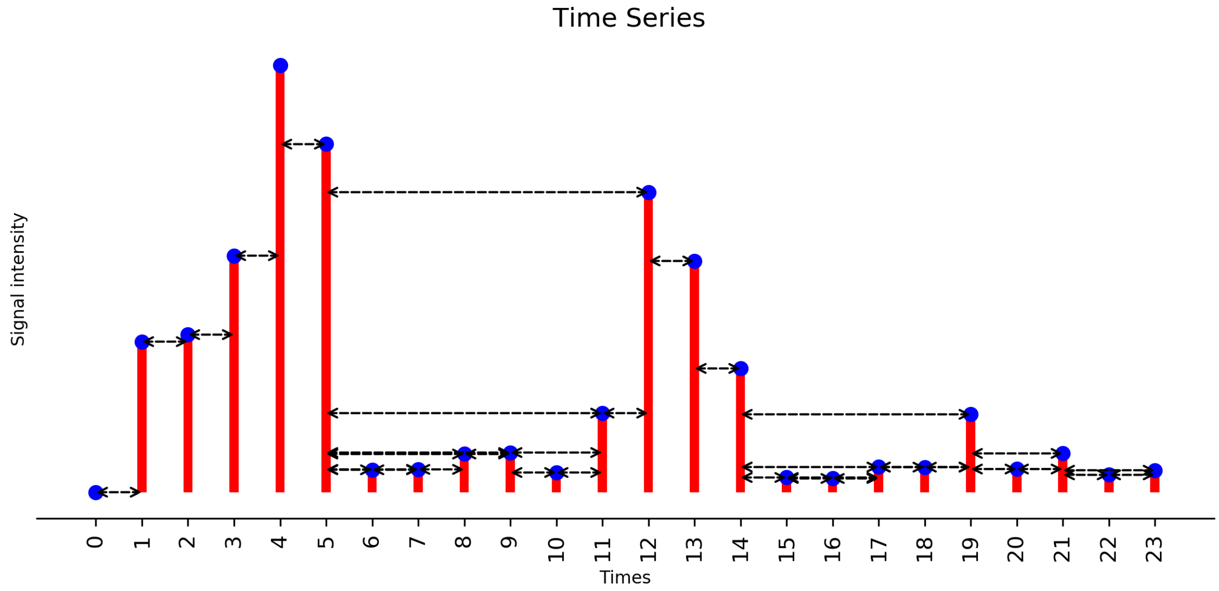

6.7 Extended DataFrame

Usage of some of the functions added to a standard DataFrame.

```
#import sys
#sys.path.append("../..")

# Import PyIOmica
import pyiomica as pio

# Import Extended DataFrame from PyIOmica
```

(continues on next page)

(continued from previous page)

```
from pyiomica.extendedDataFrame import DataFrame

# Create a simple data for testing and demonstration
df_data = DataFrame(data=pio.np.array([[0.5,2,3],
                                       [0,2,6],
                                       [7,3,0],
                                       [2,2,8],
                                       [1,pio.np.nan,pio.np.nan],
                                       [6,0,0],
                                       [0,0,0],
                                       [3,3,3.1],
                                       [3,2,pio.np.nan],
                                       [4,pio.np.nan,4]]).astype(float),
                    index=['s1','s2','s3','s4','s5','s6','s7','s8','s9','s10'],
                    columns=['c1', 'c2', 'c3'])
print(df_data, '\n')

# Remove all-zero signals from the data
df_data.filterOutAllZeroSignals(inplace=True)
print(df_data, '\n')

# Remove firt-point-zero signals from the data
df_data.filterOutReferencePointZeroSignals(inplace=True)
print(df_data, '\n')

# Remove nearly-constant signals from the data
df_data.removeConstantSignals(0.2, inplace=True)
print(df_data, '\n')

# Remove signals with >75% non-zero points
df_data.filterOutFractionZeroSignals(0.6, inplace=True)
print(df_data, '\n')

# Remove signals with >75% non-zero points
df_data.filterOutFractionMissingSignals(0.8, inplace=True)
print(df_data, '\n')

# Add a signal with zeros
df_data.loc['s11'] = [2,0,6]
print(df_data, '\n')

# Replace any zeros with np.NaN (missing)
df_data.tagValueAsMissing(inplace=True)
print(df_data, '\n')

# Replace any missing values (np.NaN) with values
df_data.tagMissingAsValue(value=0, inplace=True)
print(df_data, '\n')

# Replace any values smaller than 'a' with 'b'
df_data.tagLowValues(1., 1., inplace=True)
print(df_data, '\n')

# Calculate modified zscore (median-based) of data
df_data_zm = df_data.modifiedZScore()
print(df_data_zm, '\n')
```

(continues on next page)

(continued from previous page)

```
# Quantile normalize the data
df_data_qn = df_data.quantileNormalize()
print(df_data_qn, '\n')

# Box-cox transform data
df_data_bc = df_data.boxCoxTransform()
print(df_data_bc, '\n')

# Normalize signals to unity
df_data_un = df_data.normalizeSignalsToUnity()
print(df_data_un, '\n')
```

6.8 Time Series Categorization

```
#import sys
#sys.path.append("../..")

import pyiomica as pio

from pyiomica import categorizationFunctions as cf

if __name__ == '__main__':

    # Unzip example data
    with pio.zipfile.ZipFile(pio.os.path.join(pio.ConstantPyIOmicaExamplesDirectory,
↪'SLV.zip'), "r") as zipFile:
        zipFile.extractall(path=pio.ConstantPyIOmicaExamplesDirectory)

    # Process sample dataset SLV_Hourly1
    # Name of the fisrt data set
    dataName = 'SLV_Hourly1TimeSeries'

    # Define a directory name where results are be saved
    saveDir = pio.os.path.join('results', dataName, '')

    # Directory name where example data is (*.csv files)
    dataDir = pio.os.path.join(pio.ConstantPyIOmicaExamplesDirectory, 'SLV')

    # Read the example data into a DataFrame
    df_data = pio.pd.read_csv(pio.os.path.join(dataDir, dataName + '.csv'), index_
↪col=[0,1,2], header=0)

    # Calculate time series categorization
    cf.calculateTimeSeriesCategorization(df_data, dataName, saveDir,␣
↪NumberOfRandomSamples = 10**5)

    # Cluster the time series categorization results
    cf.clusterTimeSeriesCategorization(dataName, saveDir)

    # Make plots of the clustered time series categorization
    cf.visualizeTimeSeriesCategorization(dataName, saveDir)


    # Process sample dataset SLV_Hourly2, in the same way as SLV_Hourly1 above
```

(continues on next page)

(continued from previous page)

```
    dataName = 'SLV_Hourly2TimeSeries'
    saveDir = pio.os.path.join('results', dataName, '')
    dataDir = pio.os.path.join(pio.ConstantPyIOmicaExamplesDirectory, 'SLV')
    df_data = pio.pd.read_csv(pio.os.path.join(dataDir, dataName + '.csv'), index_
↪col=[0,1,2], header=0)
    cf.calculateTimeSeriesCategorization(df_data, dataName, saveDir,␣
↪NumberOfRandomSamples = 10**5)
    cf.clusterTimeSeriesCategorization(dataName, saveDir)
    cf.visualizeTimeSeriesCategorization(dataName, saveDir)


    # Import data storage submodule to read results of processing sample datasets SLV_
↪Hourly1 and SLV_Hourly2
    from pyiomica import dataStorage as ds

    # Use results from processing sample datasets SLV_Hourly1 and SLV_Hourly2 to␣
↪calculate "Delta"
    dataName = 'SLV_Hourly1TimeSeries'
    df_data_processed_H1 = ds.read(dataName+'_df_data_transformed', hdf5fileName=pio.
↪os.path.join('results',dataName,dataName+'.h5'))

    dataName = 'SLV_Hourly2TimeSeries'
    df_data_processed_H2 = ds.read(dataName+'_df_data_transformed', hdf5fileName=pio.
↪os.path.join('results',dataName,dataName+'.h5'))

    dataName = 'SLV_Delta'
    saveDir = pio.os.path.join('results', dataName, '')
    df_data = df_data_processed_H2.compareTwoTimeSeries(df_data_processed_H1,␣
↪compareAllLevelsInIndex=False, mergeFunction=pio.np.median).fillna(0.)
    cf.calculateTimeSeriesCategorization(df_data, dataName, saveDir,␣
↪NumberOfRandomSamples = 10**5)
    cf.clusterTimeSeriesCategorization(dataName, saveDir)
    cf.visualizeTimeSeriesCategorization(dataName, saveDir)
```

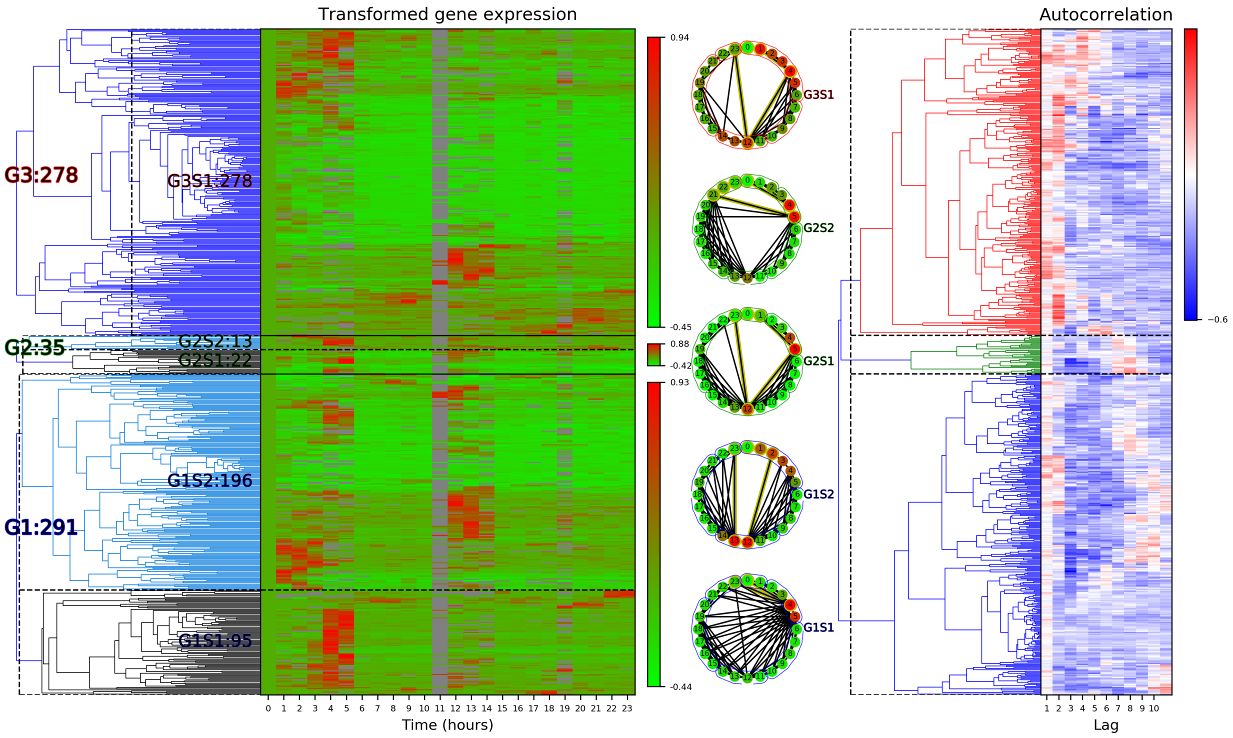
Transformed gene expression
Autocorrelation
G3:278
G3S1:278
G2:35
G2S2:13
G2S1:22
G1:291
G1S2:196
G1S1:95
G3S1
G2S2
G2S1
G1S2
G1S1
Time (hours)
Lag

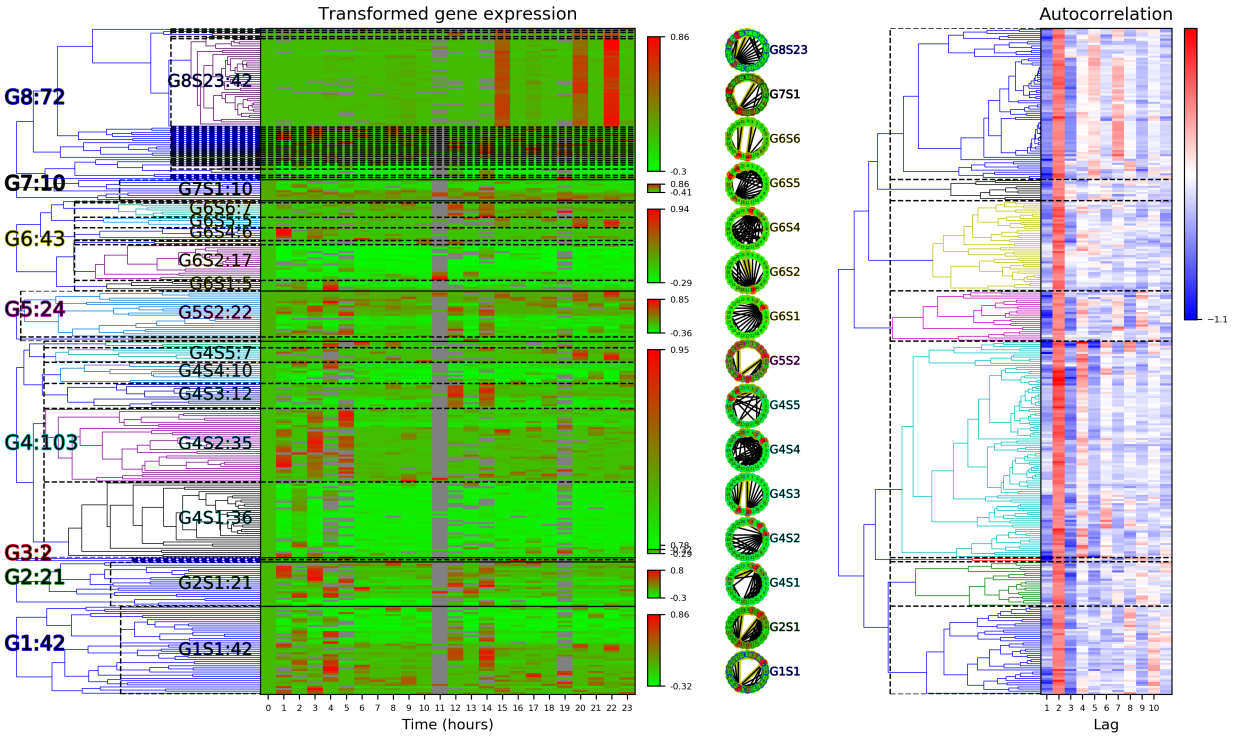
Transformed gene expression
Autocorrelation
G8:72
G8S23:42
G7:10
G7S1:10
G6:43
G6S6:7
G6S5:5
G6S4:6
G6S2:17
G6S1:5
G5:24
G5S2:22
G4S5:7
G4S4:10
G4S3:12
G4:103
G4S2:35
G4S1:36
G3:2
G2:21
G2S1:21
G1:42
G1S1:42
Time (hours)
Lag

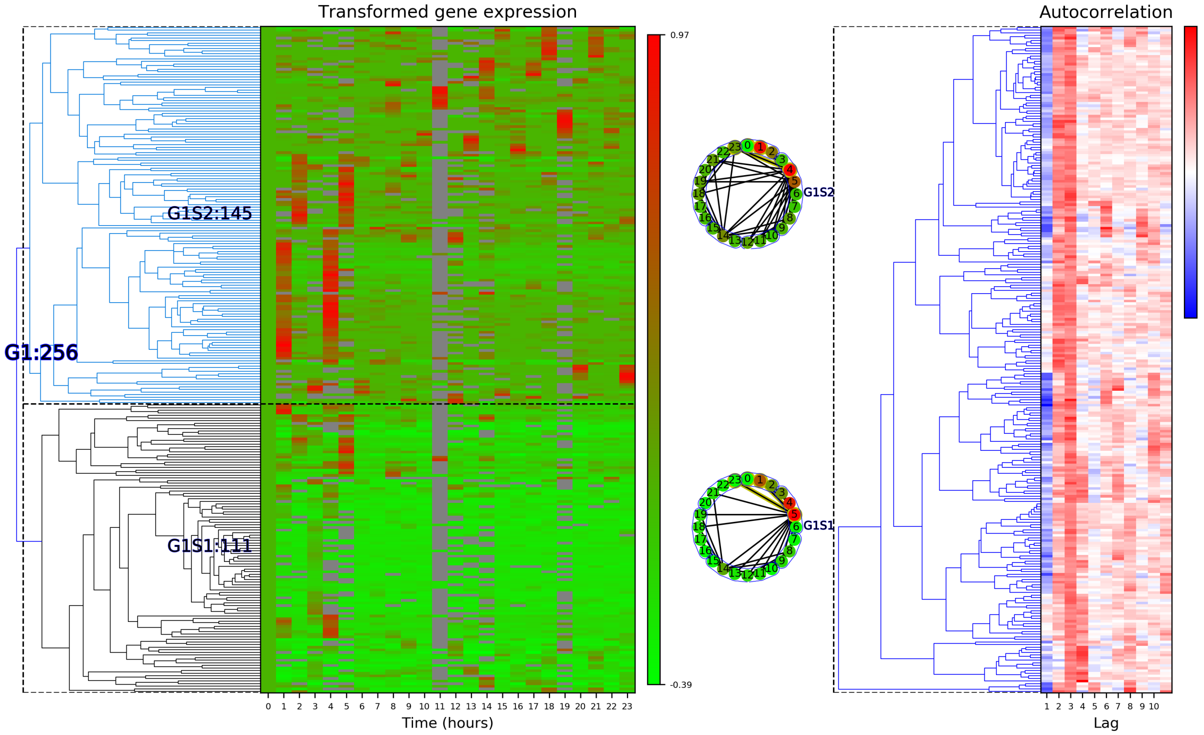
Transformed gene expression
Autocorrelation
G1:256
G1S2:145
G1S1:111
G1S2
G1S1
0.97
-0.39
Time (hours)
Lag

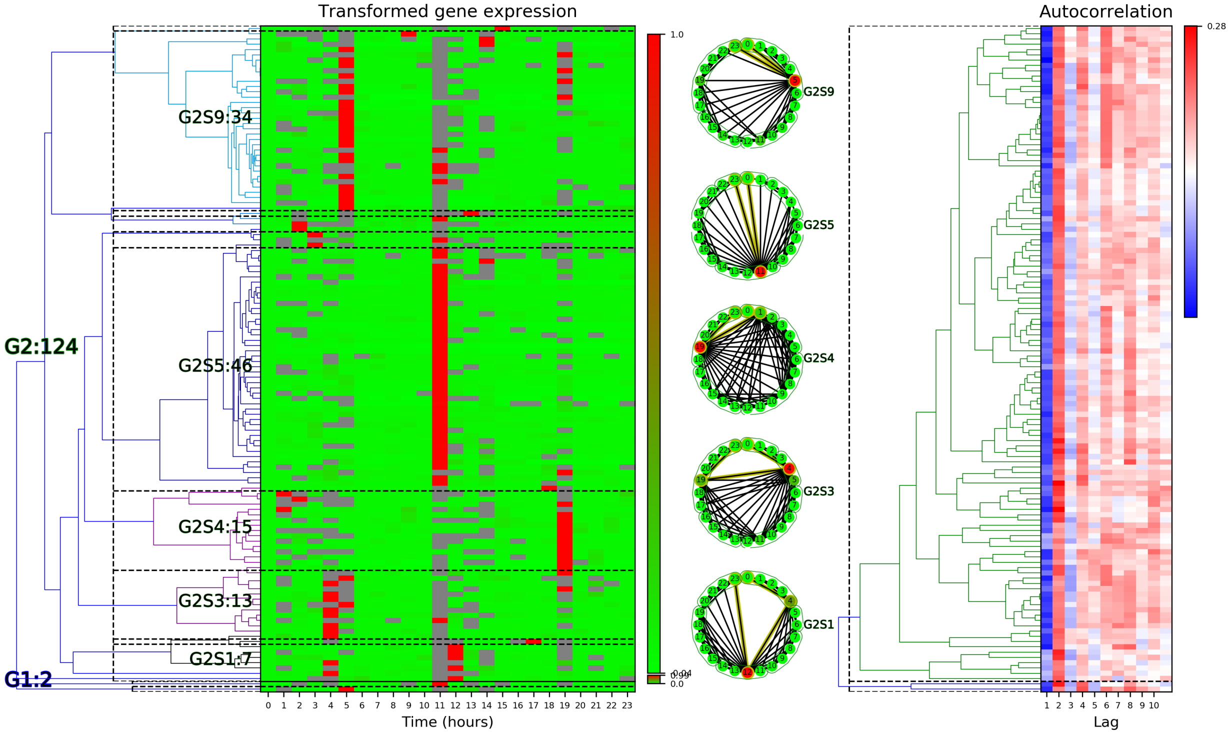
Transformed gene expression
Autocorrelation
G2:124
G1:2
G2S9:34
G2S5:46
G2S4:15
G2S3:13
G2S1:7
G2S9
G2S5
G2S4
G2S3
G2S1
1.0
0.0
0.28
Time (hours)
Lag

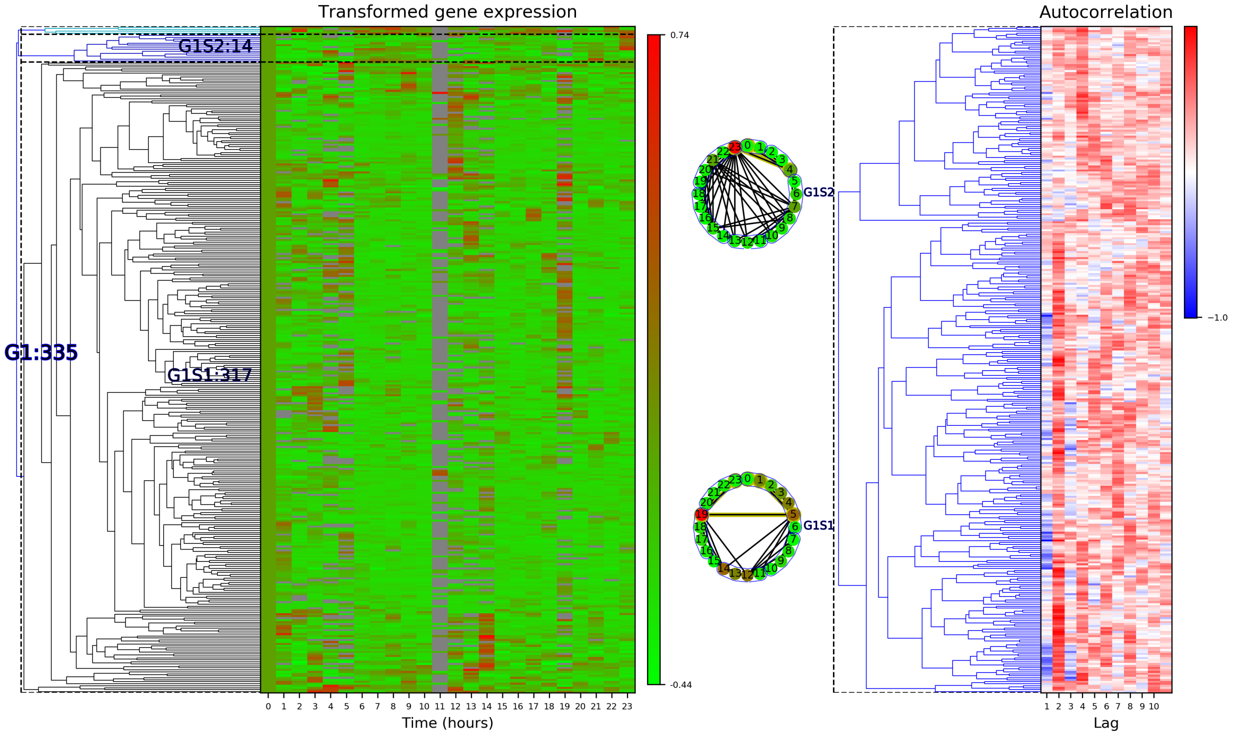


```
#import sys
#sys.path.append("../..")

import pyiomica as pio

from pyiomica import categorizationFunctions as cf
from pyiomica import dataStorage as ds
from pyiomica import enrichmentAnalyses as ea

if __name__ == '__main__':

    # Unzip example data
    with pio.zipfile.ZipFile(pio.os.path.join(pio.ConstantPyIOmicaExamplesDirectory,
↪'SLV.zip'), "r") as zipFile:
        zipFile.extractall(path=pio.ConstantPyIOmicaExamplesDirectory)

    # Name of the fisrt data set
    dataName = 'DailyTimeSeries_SLV_Protein'

    # Define a directory name where results are be saved
    saveDir = pio.os.path.join('results', dataName, '')

    # Directory name where example data is (*.csv files)
    dataDir = pio.os.path.join(pio.ConstantPyIOmicaExamplesDirectory, 'SLV')

    # Read the example data into a DataFrame
    df_data = pio.pd.read_csv(pio.os.path.join(dataDir, dataName + '.csv'), index_
↪col=[0,1], header=0)
```

(continues on next page)

(continued from previous page)

```
    # Calculate time series categorization
    cf.calculateTimeSeriesCategorization(df_data, dataName, saveDir, NumberOfRandomSamples = 10**5, referencePoint=2, preProcessData=False)

    # Cluster the time series categorization results
    cf.clusterTimeSeriesCategorization(dataName, saveDir)

    # Make plots of the clustered time series categorization
    cf.visualizeTimeSeriesCategorization(dataName, saveDir)

    # Do enrichment GO and KEGG analysis on LAG1 results
    LAG1 = ds.read('results/DailyTimeSeries_SLV_Protein/consolidatedGroupsSubgroups/DailyTimeSeries_SLV_Protein_LAG1_Autocorrelations_GroupsSubgroups')
    ea.ExportEnrichmentReport(ea.GOAnalysis(LAG1), AppendString='GO_LAG1', OutputDirectory='results/DailyTimeSeries_SLV_Protein/')
    ea.ExportEnrichmentReport(ea.KEGGAnalysis(LAG1), AppendString='KEGG_LAG1', OutputDirectory='results/DailyTimeSeries_SLV_Protein/')
```

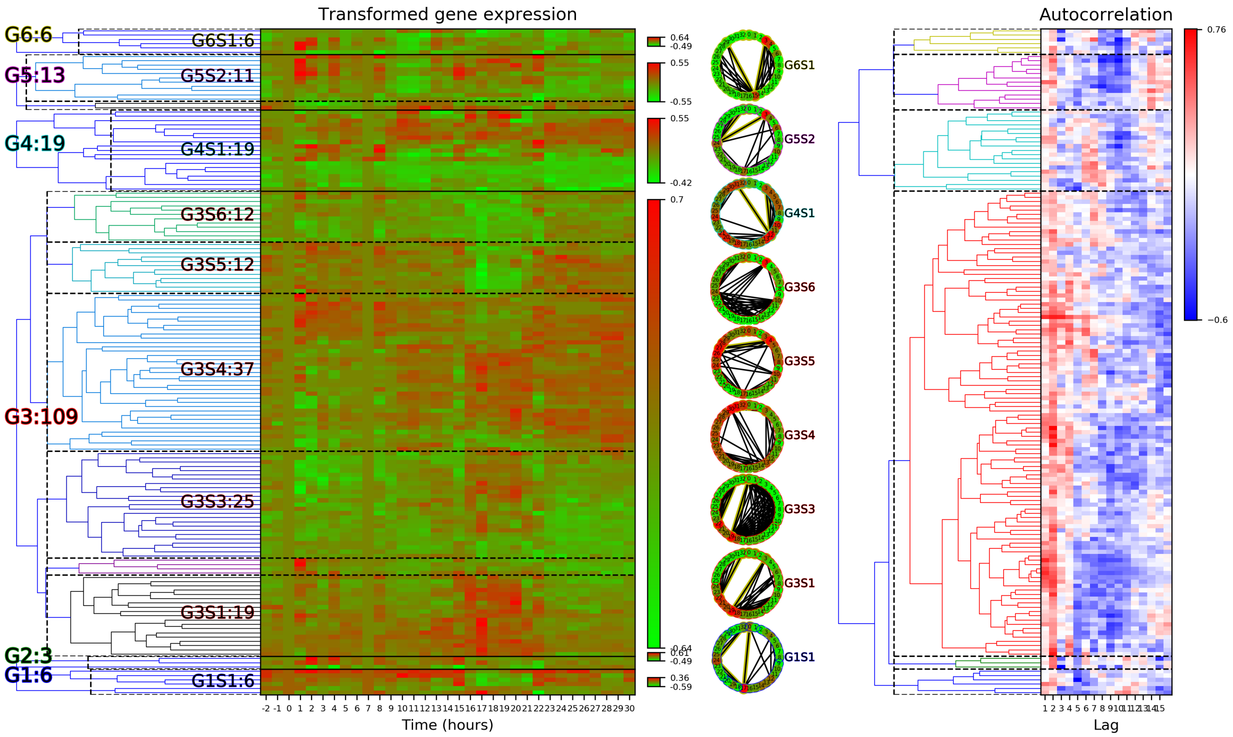

CHAPTER 7

Indices and tables

- genindex
- modindex
- search

Python Module Index

p

pyiomica.categorizationFunctions, 9
pyiomica.clusteringFunctions, 26
pyiomica.enrichmentAnalyses, 11
pyiomica.extendedDataFrame, 19
pyiomica.globalVariables, 7
pyiomica.utilityFunctions, 35
pyiomica.visibilityGraphAuxiliaryFunctions, 29
pyiomica.visualizationFunctions, 30

Index

Symbols

__init__() (*DataFrame method*), 19

A

addColorbarToFigure() (*in module pyiomica.visualizationFunctions*), 34
addVisibilityGraph() (*in module pyiomica.visualizationFunctions*), 31

B

BenjaminiHochbergFDR() (*in module pyiomica.enrichmentAnalyses*), 19
boxCoxTransform() (*DataFrame method*), 22

C

calculateTimeSeriesCategorization() (*in module pyiomica.categorizationFunctions*), 9
clusterTimeSeriesCategorization() (*in module pyiomica.categorizationFunctions*), 10
compareTimeSeriesToPoint() (*DataFrame method*), 24
compareTwoTimeSeries() (*DataFrame method*), 24
ConstantGeneDictionary (*in module pyiomica.globalVariables*), 8
ConstantPyIOmicaDataDirectory (*in module pyiomica.globalVariables*), 7
ConstantPyIOmicaExamplesDirectory (*in module pyiomica.globalVariables*), 7
ConstantPyIOmicaExampleVideosDirectory (*in module pyiomica.globalVariables*), 7
createDirectories() (*in module pyiomica.utilityFunctions*), 35
createReverseDictionary() (*in module pyiomica.utilityFunctions*), 35

D

DataFrame (*class in pyiomica.extendedDataFrame*), 19

E

exportClusteringObject() (*in module pyiomica.clusteringFunctions*), 28
ExportEnrichmentReport() (*in module pyiomica.enrichmentAnalyses*), 18

F

filterOutAllZeroSignals() (*DataFrame method*), 20
filterOutFractionMissingSignals() (*DataFrame method*), 21
filterOutFractionZeroSignals() (*DataFrame method*), 20
filterOutReferencePointZeroSignals() (*DataFrame method*), 21

G

GeneTranslation() (*in module pyiomica.enrichmentAnalyses*), 15
get_n_clusters_from_linkage_Elbow() (*in module pyiomica.clusteringFunctions*), 26
get_n_clusters_from_linkage_Silhouette() (*in module pyiomica.clusteringFunctions*), 27
getAdjacencyMatrixOfHVG (*in module pyiomica.visibilityGraphAuxiliaryFunctions*), 29
getAdjacencyMatrixOfHVGbyNUMPY() (*in module pyiomica.visibilityGraphAuxiliaryFunctions*), 30
getAdjacencyMatrixOfNVG (*in module pyiomica.visibilityGraphAuxiliaryFunctions*), 29
getAdjacencyMatrixOfNVGbyNUMPY() (*in module pyiomica.visibilityGraphAuxiliaryFunctions*), 29
getCommunitiesOfTimeSeries() (*in module pyiomica.clusteringFunctions*), 28
getEstimatedNumberOfClusters() (*in module pyiomica.clusteringFunctions*), 26
GetGeneDictionary() (*in module pyiomica.enrichmentAnalyses*), 12

getGroupingIndex() (in module pyiomica.clusteringFunctions), 27
getLobmScarglePeriodogramOfDataframe() (in module pyiomica.extendedDataFrame), 25
getRandomAutocorrelations() (in module pyiomica.extendedDataFrame), 25
getRandomPeriodograms() (in module pyiomica.extendedDataFrame), 25
getRandomSpikesCutoffs() (in module pyiomica.extendedDataFrame), 25
GOAnalysis() (in module pyiomica.enrichmentAnalyses), 14
GOAnalysisAssigner() (in module pyiomica.enrichmentAnalyses), 13

I

imputeMissingWithMedian() (DataFrame method), 24
internalAnalysisFunction() (in module pyiomica.enrichmentAnalyses), 12

K

KEGGAnalysis() (in module pyiomica.enrichmentAnalyses), 16
KEGGAnalysisAssigner() (in module pyiomica.enrichmentAnalyses), 15
KEGGDictionary() (in module pyiomica.enrichmentAnalyses), 16

M

makeClusteringObject() (in module pyiomica.clusteringFunctions), 28
makeDataHistograms() (in module pyiomica.visualizationFunctions), 30
makeDendrogramHeatmapOfClusteringObject() (in module pyiomica.visualizationFunctions), 34
makeLombScarglePeriodograms() (in module pyiomica.visualizationFunctions), 31
makePlotOfPeak() (in module pyiomica.visualizationFunctions), 34
makeVisibilityBarGraph() (in module pyiomica.visualizationFunctions), 33
makeVisibilityGraph() (in module pyiomica.visualizationFunctions), 32
MassDictionary() (in module pyiomica.enrichmentAnalyses), 18
MassMatcher() (in module pyiomica.enrichmentAnalyses), 18
mergeDataframes() (in module pyiomica.extendedDataFrame), 24
modifiedZScore() (DataFrame method), 22

N

normalizeSignalsToUnity() (DataFrame method), 23

O

OBOGODictionary() (in module pyiomica.enrichmentAnalyses), 12
obtainConstantGeneDictionary() (in module pyiomica.enrichmentAnalyses), 13

P

PackageDirectory (in module pyiomica.globalVariables), 7
printPackageGlobalDefaults (in module pyiomica.globalVariables), 7
pyiomica.categorizationFunctions (module), 9
pyiomica.clusteringFunctions (module), 26
pyiomica.enrichmentAnalyses (module), 11
pyiomica.extendedDataFrame (module), 19
pyiomica.globalVariables (module), 7
pyiomica.utilityFunctions (module), 35
pyiomica.visibilityGraphAuxiliaryFunctions (module), 29
pyiomica.visualizationFunctions (module), 30

Q

quantileNormalize() (DataFrame method), 23

R

readMathIOmicaData() (in module pyiomica.utilityFunctions), 35
removeConstantSignals() (DataFrame method), 22
runCPUs() (in module pyiomica.utilityFunctions), 35
runForClusterNum() (in module pyiomica.clusteringFunctions), 27

S

saveFigure() (in module pyiomica.visualizationFunctions), 30

T

tagLowValues() (DataFrame method), 22
tagMissingAsValue() (DataFrame method), 21
tagValueAsMissing() (DataFrame method), 21

V

visualizeTimeSeriesCategorization() (in module pyiomica.categorizationFunctions), 10
